# Estimation of Activity Related Energy Expenditure and Resting Metabolic Rate in Freely Moving Mice from Indirect Calorimetry Data

**DOI:** 10.1371/journal.pone.0036162

**Published:** 2012-05-04

**Authors:** Jan Bert Van Klinken, Sjoerd A. A. van den Berg, Louis M. Havekes, Ko Willems Van Dijk

**Affiliations:** 1 Department of Human Genetics, Leiden University Medical Center, Leiden, The Netherlands; 2 Department of General Internal Medicine, Leiden University Medical Center, Leiden, The Netherlands; 3 Department of Cardiology, Leiden University Medical Center, Leiden, The Netherlands; 4 Netherlands Organisation for Applied Scientific Research-Quality of Life, Leiden, The Netherlands; Institut Pluridisciplinaire Hubert Curien, France

## Abstract

Physical activity (PA) is a main determinant of total energy expenditure (TEE) and has been suggested to play a key role in body weight regulation. However, thus far it has been challenging to determine what part of the expended energy is due to activity in freely moving subjects. We developed a computational method to estimate activity related energy expenditure (AEE) and resting metabolic rate (RMR) in mice from activity and indirect calorimetry data. The method is based on penalised spline regression and takes the time dependency of the RMR into account. In addition, estimates of AEE and RMR are corrected for the regression dilution bias that results from inaccurate PA measurements. We evaluated the performance of our method based on 500 simulated metabolic chamber datasets and compared it to that of conventional methods. It was found that for a sample time of 10 minutes the penalised spline model estimated the time-dependent RMR with 1.7 times higher accuracy than the Kalman filter and with 2.7 times higher accuracy than linear regression. We assessed the applicability of our method on experimental data in a case study involving high fat diet fed male and female C57Bl/6J mice. We found that TEE in male mice was higher due to a difference in RMR while AEE levels were similar in both groups, even though female mice were more active. Interestingly, the higher activity did not result in a difference in AEE because female mice had a lower caloric cost of activity, which was likely due to their lower body weight. In conclusion, TEE decomposition by means of penalised spline regression provides robust estimates of the time-dependent AEE and RMR and can be applied to data generated with generic metabolic chamber and indirect calorimetry set-ups.

## Introduction

In the last few decades a great deal of effort has been devoted to measuring energy expenditure in humans and animal models, with the aim of uncovering potential causes for obesity. Activity-related energy expenditure is the most variable component of total energy expenditure [1], [2] and has been shown to play a key role in achieving energy balance in humans [3], [4] and rodents [5], [6]. The resting component of TEE, or resting metabolic rate, consists of the basal metabolic rate, which is the minimum level of energy required by the body to sustain vital functions, and the thermic effect of food, which is the energy needed for the digestion of food [7], [8]. In addition, at temperatures outside the thermoneutral range, a substantial part of RMR comprises energy expenditure involved in thermoregulation [9]. Low levels of RMR have been identified as a risk factor for future weight gain in Pima Indians [10], [11] and adaptations in RMR have been linked to weight regain after extensive weight loss in rats [12].

Since subtle differences in daily energy expenditure can lead to substantial changes in weight when integrated over extended periods of time, it is vital to have accurate estimates of AEE and RMR in order to gain a better and more quantitative understanding of their role in the development of overweight. Estimation of the contribution of AEE and RMR to TEE in freely moving rodents, however, has proven to be difficult. The standard approach is to simultaneously monitor physical activity and TEE of an animal in a metabolic chamber employing indirect calorimetry. Subsequently, the contribution of AEE and RMR to TEE is determined by linear regression [6] or by taking the TEE that is not associated with activity [5], [12], [13]. However, since these approaches do not take into account that the RMR varies with time – e.g. due to diurnal variations, the thermic effect of food, or nervous and hormonal changes – the estimates of the AEE and RMR that are thus obtained are relatively inaccurate.

A significant improvement with respect to these methods has been proposed by Even *et al.* [14], who showed that time-dependent estimates of RMR can be obtained from indirect calorimetry data by means of Kalman filtering. Although this method gives reliable and reproducible results in mice [15] and rats [16], specialised equipment and high time resolution monitoring of activity and respiratory gas exchange are needed for the Kalman filter to give optimal results.

Here, we propose a computational method that allows to determine the RMR and AEE in mice from data generated by generic, commercially available metabolic chambers employing indirect calorimetry. The method is based on modelling the time variations in RMR by means of penalised cubic spline functions and includes a correction for the bias introduced by inaccurate measurements of physical activity. Both features enable the use of respiratory gas exchange data that has been generated at low time resolutions and with infrared beam activity monitors, as they are provided by conventional systems of metabolic chambers for rodents. We evaluated the performance of our method on simulated indirect calorimetry data and show that it is robust to changes in sample time resolution and measurement error in PA. In addition, we demonstrate its applicability in a case study of high fat diet fed male and female C57Bl/6J mice.

## Results

### Penalised Spline Regression

We here give a short description of the penalised spline (P-spline) regression model to decompose the TEE; for a more extensive discussion, see Methods. Our method is based on the assumption that the slow time variations in the RMR can be modelled by a set of spline functions and that the AEE linearly correlates with the intensity of PA. Estimation of the spline coefficients and of the caloric cost of activity (CCA), i.e. the scale factor of AEE to PA, occurs by minimising the residual sum of squares of the model with respect to the time sequence of TEE measurements. Since gas diffusion effects deform the time dependency of the measured TEE, this deformation is modelled by means of linear compartments and applied to the PA time sequence. Variability in the spline coefficients is diminished by penalising the first order derivative of the spline function, thus ensuring that smooth results are obtained and no overfitting occurs to measurement noise [17]. The degree of penalisation is determined from the data using the generalised cross validation criterion [18]. In order to account for the regression dilution bias that is introduced into the CCA estimate by inaccuracies in the PA measurements, a corrected estimator was devised based on a multiplicative errors-in-variables model.

Since the P-spline model is based on the assumption that the relation between PA and AEE is linear, this must be ascertained by inspecting the scatterplot of the TEE and PA. If the relation is nonlinear, the measured PA has to be preprocessed accordingly. Selection of the parameters of the preprocessing function can be based on minimisation of the residual sum of squares of the P-spline model. See Supplementary Text S2 for details.

### Validation Study

The P-spline estimation method was validated on a set of simulated high time resolution metabolic chamber datasets. An experimental high time resolution metabolic chamber dataset (TEE and PA measured every 10 s) served as basis for providing realistic simulation parameters and to verify the results from the simulation study. Performance of the P-spline method was determined based on the accuracy of the RMR estimate and on its robustness to low sample rates and large chamber sizes. Overall performance was compared to that of three other methods: linear regression, averaging TEE for periods of zero activity and Kalman filtering (Supplementary Text S3). The value of including TEE decomposition in indirect calorimetry data analysis was assessed by applying the P-spline model to data from a case study in which male and female C57Bl/6J mice had been put on a 10 week high fat diet. For details regarding the set-up of the validation study, see Methods.

### Performance Evaluation

**Figure 1:**
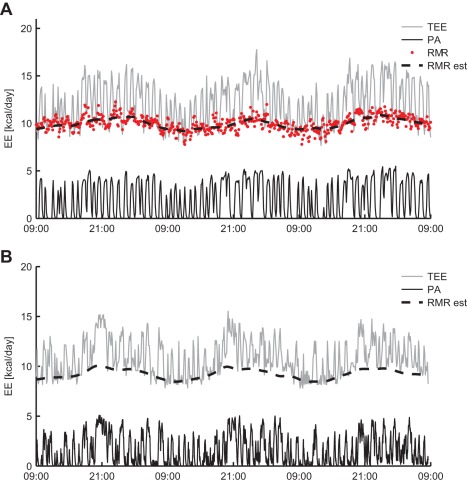
Estimation of resting metabolic rate in experimental and simulated data. Mouse metabolic chamber datasets were simulated, consisting of the total energy expenditure (TEE), resting metabolic rate (RMR) and physical activity (PA) (**A**). The simulated data shows to be similar to the experimental data (**B**), exhibiting a diurnal rhythm in activity patterns and RMR, and high frequency time variations in the TEE that are due to PA. Simulated datasets were used to evaluate the accuracy of the estimated RMR time series (RMR est) by means of penalised spline regression. For details regarding the simulation procedure, see the Methods section and Supplementary Text 4.

**Figure 2:**
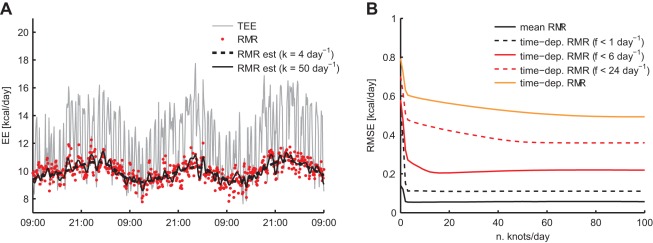
Knot number selection. The time variations in the RMR that are estimated by the penalised spline model depend on the number of knots used in the spline function: 4 knots/day are sufficient to capture the diurnal rhythm in the RMR, whereas more knots are needed to estimate faster time variations (**A**). Calculating the root mean square estimation error (RMSE) for a range of frequency components in the RMR shows that more accurate estimates of high frequency components are obtained when the knot number is increased (**B**). Roughly, 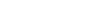 are needed to estimate frequency components in the RMR of up to 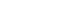. However, higher frequency components are estimated with a relatively larger error.

A total of 500 metabolic chamber datasets were simulated to determine the accuracy of the P-spline regression model in estimating RMR. The simulated datasets consisted of TEE, RMR and PA and resembled experimental data with respect to typical activity patterns and circadian and ultradian variations in RMR (Fig. 1). First the influence of the spline function’s knot number on the RMR estimate was evaluated. It followed that with low knot numbers only slowly varying components of RMR could be estimated accurately, whereas the knot number needed to be increased to estimate the higher frequency components (Fig. 2). Approximately, with *k* knots per day the time variations in RMR with frequencies up to 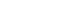 were estimated optimally. It also followed from these results that higher frequency components in RMR could not be estimated accurately; therefore, in the remainder of this study we quantified the accuracy of time-dependent RMR estimation as the error in estimating the frequency components in RMR under 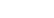 (see Methods).

**Figure 3:**
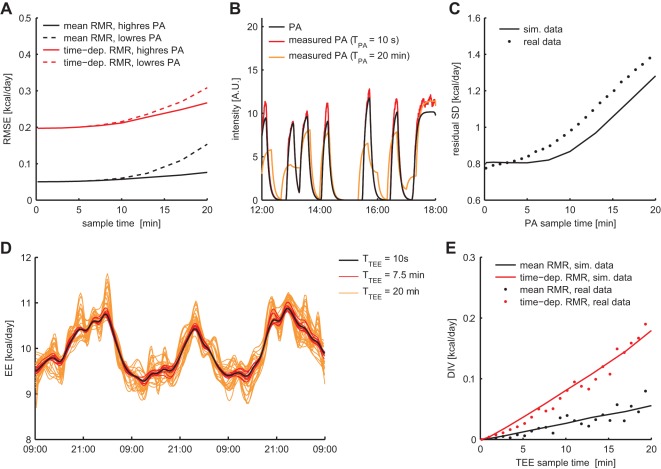
Estimation accuracy of the penalised spline model and dependency on the sample rate. The accuracy of the penalised spline model in estimating the average and time-dependent RMR deteriorates when TEE and PA are sampled at a lower rate (**A**). This effect is less strong if TEE has been sampled at a low rate but PA at a high rate. The effect of sampling PA at a low rate is that fast transitions between active and inactive periods are not rendered faithfully (**B**). These inaccuracies result in a larger unexplained variance of the fitted penalised spline model on both the simulated and experimental data (**C**). The effect of sampling TEE at a low rate on the time-dependent RMR estimates is illustrated by transforming a single high resolution dataset into *N* low resolution datasets, and superimposing the *N* different RMR estimates (**D**). When the TEE time series are downsampled by a factor *N* = 120, corresponding to a sample time of 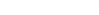, there is considerably more variation in the RMR estimates than with a downsampling factor of *N* = 45 (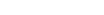). The variability in the RMR estimates (the downsampling induced variability; DIV) showed a linear dependency on 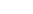 for simulated and experimental data (**E**).

The performance of the P-spline model was evaluated by considering the estimation error of the average and time-dependent RMR, for varying sample times *T* of the TEE and PA. When TEE and PA were sampled at the same rate, the Root Mean Square Error (RMSE) of the average RMR estimate increased monotonically from 0.5% for 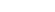 to 1.5% for 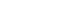 (relative to the true average RMR of 10 kcal/day). The RMSE of the time-dependent estimate increased from 2.0% for 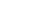 to 3.1% for 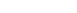 (Fig. 3A). Estimation accuracy improved when PA was sampled at 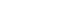 and TEE at a variable rate: for 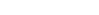, the RMSE of the average and time-dependent RMR estimate were 0.76% and 2.7% respectively.

The origin of the estimation error that is introduced when PA is sampled at a low rate becomes evident when comparing the time sequence of the actual PA and the measured PA at high and low sample rates (Fig. 3B). When PA is measured with a sample time of 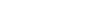, there is a larger deviation from the actual PA than when it is sampled with 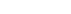. This effect is illustrated by the dependency of the residual variance of the P-spline model on 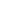, which shows that there is more unexplained variance for higher 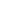 (Fig. 3C). Interestingly, the effect is even stronger on the high resolution experimental dataset, which suggests that nonlinearity of the PA-AEE relation in combination with a low sample rate adds to the unexplained variance.

We quantified the uncertainty in the RMR estimate due to low sample times as the downsampling induced variability (DIV), which we defined as the variability between estimated RMR time sequences when based on subsequent time points (Fig. 3D). On the simulated data the relative DIV increased linearly to 0.56% for the average RMR and to 1.8% for the time-dependent RMR, for 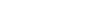 (Fig. 3E). The same trend in DIV was found for the high time resolution experimental dataset, showing that the variance in the estimation error of the P-spline method was similar for simulated and experimental data.

**Figure 4:**
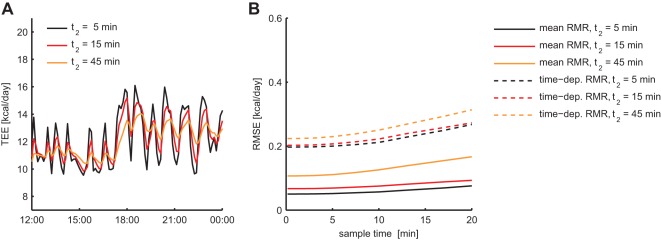
Dependency of estimation accuracy on chamber size to flow rate ratio. Increasing the chamber size to flow rate ratio (the washout time 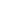), the high frequency variations in TEE that are caused by PA are attenuated by the filtering effect of gas diffusion through the metabolic chamber (**A**). Consequently, accurate TEE decomposition becomes more difficult, as demonstrated by the increase in estimation error of the average and time-dependent RMR for larger 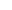 (**B**).

Simulating data with a higher chamber size to flow rate ratio – which we here refer to as the washout time 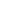 (see Methods) – the high frequency variations in TEE due to PA were notably reduced (Fig. 4A). The accuracy of average RMR estimation decreased slightly when 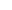 was increased from 5 to 15 min, whereas it declined considerably for 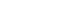 (Fig. 4B). Increasing 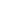 also had a negative effect on the accuracy of the time-dependent RMR estimation, but this effect was less strong due to the fact that also high frequency variations in RMR had been filtered out. These findings show that less accurate results are obtained when either the chamber volume is increased or the flow rate is decreased. Importantly, it was found that for each washout time 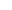 shorter sampling times 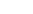 allowed for more accurate estimates. This implies that measuring respiratory gas exchange with short sample times is advantageous for TEE decomposition, even when the chamber size to flow rate ratio is large.

### Comparison with Other Methods

**Figure 5:**
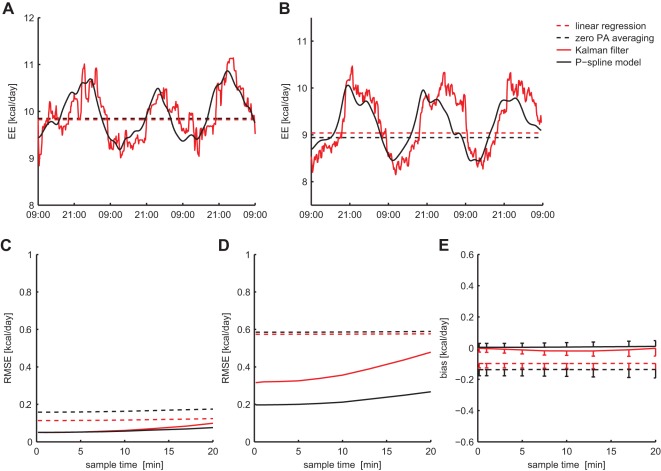
Performance comparison of estimation methods. Estimated RMR time series by means of linear regression, TEE averaging for zero activity, Kalman filtering and penalised spline modelling are shown for a typical simulated (**A**) and experimental (**B**) dataset. The overall estimation accuracy in average RMR (**C**) and time-dependent RMR (**D**) was calculated based on 500 simulated datasets (expressed as Root Mean Square Error). For the estimation error in the average RMR the bias-variance decomposition was calculated to gain more insight in the origin of the error (**E**). Error bars indicate half the standard deviation.

The performance of the P-spline regression model was compared to that of the linear regression model, the method of averaging TEE for periods of zero activity and Kalman filtering. The RMR time sequences that were estimated by each method gave similar results on simulated and experimental data (Fig. 5A,B), suggesting that the time variation in the RMR had been simulated in a realistic fashion. The estimates from the linear regression model and the TEE averaging for zero activity method were constant over time, making thus a relatively large error in estimating the time-dependent RMR. The estimate of the Kalman filter and P-spline model showed to successfully follow the evolution of the RMR, although it exhibited more variability.

The performance of the four estimation methods was assessed by considering the estimation error of average RMR (Fig. 5C) and time-dependent RMR (Fig. 5D), for varying sample times of the TEE. Since we focused on estimating the low frequency components in the RMR, the high frequency variability in the Kalman filter estimate was filtered out. To obtain an insight into the origin of the differences in estimation accuracy, the estimation error of average RMR was decomposed into a bias and variance term (Fig. 5E). The P-spline model and Kalman filter performed equally well in estimating the average RMR for 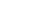 under 10 min, while the Kalman filter was little less robust for lower sample rates, amounting to a RMSE of 1.0% at 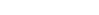. The bias in the average RMR estimate for both methods was negligible with respect to the variance component. The linear regression and zero activity TEE averaging method had considerably larger errors, which was mainly caused by the larger bias. For the estimation of time-dependent RMR, it was found that the accuracy of the P-spline model was considerably higher than that of the other methods: linear regression and averaging TEE for zero activity had a relative error of 5.8% for all sample rates, while the estimation error of the Kalman filter ranged between 3.2% for 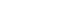 and 4.8% for 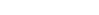.

### Case Study

**Figure 6:**
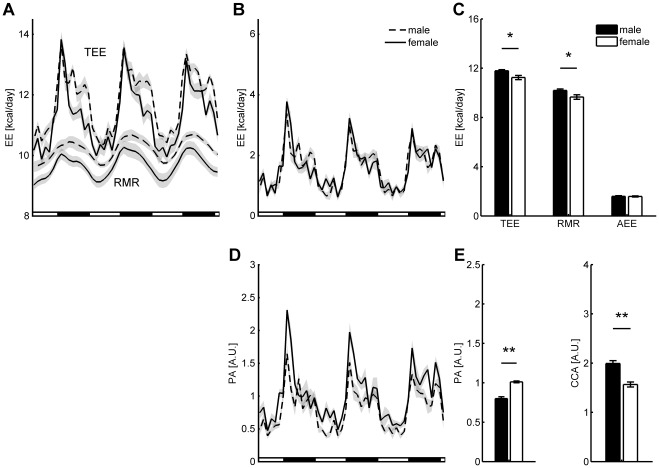
Case study: energy expenditure and activity of male and female mice after a 10 week high fat diet. Time-dependent group averages are shown of TEE and RMR (**A**) and AEE (**B**) for male and female mice. Lines indicate group averages and gray bands represent standard error of the mean; black-white bars indicate the dark-light periods. The P-spline model contained 15 knots/day. The TEE of male mice was higher during most of the day, except for the start of the dark period. The RMR of male mice was higher than that of female mice during each period of the day, whereas AEE was overall similar, except for the start of the dark period. Group differences were also present in average daily TEE and RMR, whereas no differences were found in average daily AEE (**C**). Time-dependent group averages of spontaneous PA show that female mice were more active, especially during the first phase of the dark period (**D**). The reason that the higher activity of female mice did not engender a difference in energy expenditure is that the caloric cost of activity (CCA) was lower in this group (**E**). (error bars represent standard error of the mean; ^*^ *P*<0.05, ^**^ *P*<0.001).

The P-spline regression model was applied to data from an indirect calorimetry experiment involving high fat diet fed male and female mice. Average daily TEE in male mice was found to be higher, while levels of PA were lower (Fig. 6C,E). From the P-spline model it followed that the higher TEE in male mice was explained by the fact that their RMR was higher, while average AEE was the same in both groups. The reason that the higher level of PA in female mice did not give rise to a higher AEE was that male mice had a significantly higher cost of activity than female mice (Fig. 6E). Inspecting the group averages of the time-dependent RMR and AEE it followed that male mice had a higher level of RMR during each period of 24 h, while AEE was overall similar (Fig. 6A,B). The time-dependent RMR of both groups showed a clear circadian rhythm; no difference was found in the peak-to-peak amplitude in RMR between groups (Tab. 1).

**Table 1:** Comparison of metabolic parameters of male and female C57Bl/6J mice after a 10 week high fat diet.

	Male (*n* = 8)	Female (*n* = 7)	*P*-value
Body weight [g]	36.79±4.49	23.66±1.20	4.1⋅10^–5^
TEE [kcal/day]	11.78±0.26	11.24±0.45	0.020
RMR [kcal/day]	10.19±0.33	9.66±0.49	0.034
AEE [kcal/day]	1.59±0.15	1.58±0.13	0.91
CCA [A.U.]	1.99±0.16	1.57±0.13	7.3⋅10^–5^
PA [A.U.]	0.80±0.06	1.01±0.03	6.5⋅10^–6^
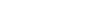 [kcal/day]	0.87±0.31	1.00±0.34	0.44

Table shows body weight, total energy expenditure (TEE), resting metabolic rate (RMR), activity related energy expenditure (AEE), caloric cost of activity (CCA), physical activity (PA) and the peak to peak amplitude in the RMR for male and female mice. Interestingly, even though PA was higher in female mice, this did not engender a difference in AEE since CCA was higher in male mice.

Since male mice were significantly heavier than female mice, an important part of the observed difference in energy expenditure was likely due to the difference in body weight. A widely used statistical technique to separate group effects on energy expenditure from body weight effects is analysis of covariance [19]–[21]. Unfortunately, analysis of covariance was not possible on our data since the within group variance in the body weight was too small, which caused the corresponding covariate term to be non-significant. Therefore, we merged both groups and performed regression analysis for each metabolic parameter with body weight (Tab. 2). It was found that body weight correlated positively with TEE (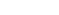), RMR (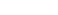) and caloric cost of activity (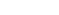), indicating that heavier animals needed more energy to move. Interestingly, activity levels were inversely correlated with body weight (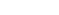), suggesting that heavier animals are less prone to physical activity.

**Table 2:** Correlation of metabolic parameters with body weight (*n* = 15).

	intercept±SE	slope±SE	*r* ^2^	*P*-value
TEE [kcal/day]	10.29±0.38	0.0406±0.0120	0.47	5.0⋅10^–3^
RMR [kcal/day]	8.655±0.425	0.0420±0.0135	0.43	8.2⋅10^–3^
AEE [kcal/day]	1.631±0.155	–0.0015±0.0049	0.01	0.77
CCA [A.U.]	1.040±0.216	0.0246±0.0069	0.50	3.4⋅10^–3^
PA [A.U.]	1.309±0.076	–0.0134±0.0024	0.70	9.2⋅10^–5^

Table shows regression coefficients (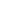 standard error; SE) of the linear correlation between body weight and total energy expenditure (TEE), resting metabolic rate (RMR), activity related energy expenditure (AEE), caloric cost of activity (CCA) and physical activity (PA). Male and female mice were merged into a single group.

## Discussion

In this work, we have shown that the time dynamics present in data from metabolic chamber experiments can be exploited to decompose the total energy expenditure into a resting and activity related component. We propose a method to decompose the TEE in a time dynamic fashion that is based on penalised spline (P-spline) regression. Penalised splines have found wide application in the field of smoothing and semiparametric regression [17], [22], and have a strong base in statistics because of their link to mixed-effects models. Here, the smoothness of the spline functions was exploited to model the slow time variations in the RMR, while the penalisation factor served to prevent overfitting to noise. In addition, we modelled inaccuracies in the activity measurements with a multiplicative errors-in-variables model, which reduced the bias due to regression dilution.

### Method Validation

We validated our method by determining its accuracy in estimating the RMR on 500 simulated metabolic chamber datasets. The simulation parameters were based on experimental data in order to ensure realistic results. It was found that the P-spline regression model was particularly accurate in estimating the average RMR and the low frequency components in the RMR, whereas the higher frequency components were more difficult to estimate due to noise interference. Investigating the effect of the number of knots on the RMR estimate, it was found that a larger number of knots was required to estimate the higher frequency components. Importantly, no other model parameters were needed to be chosen *a priori* by the user to obtain optimal results.

The sample time with which the TEE and PA were measured was found to be a major determinant of the estimation accuracy of the P-spline model. With a lower sample time less datapoints were obtained which caused a higher degree of uncertainty in the regression estimate. Similar findings have been reported by Cooper and Withers, who have shown that the sample time can affect estimates of the basal metabolic rate [23]. An advantage of the P-spline model is that it, as a result of its non-causal design, can exploit the entire TEE and PA sequence for estimating the RMR at each single time point, making it relatively robust to low sample rates.

Interestingly, the simulation study showed that the estimation accuracy was improved when PA was sampled at a higher rate than the TEE. The reason for this is that when both 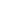 and 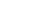 are large, then infrared beam breaks are binned into relatively long time intervals, which means that information is lost about when a beam break actually occurred. As a result, the time dynamics in the PA are measured with less precision. In contrast, when PA is measured at a higher rate than the TEE, then it is possible to convolve the PA time series with the gas diffusion impulse response 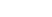 before it is downsampled to the time resolution of the TEE. The resulting time series correlate much better with the TEE as is illustrated in Fig. 3C, and therefore yield a better estimate of the AEE and RMR. Importantly, since the PA sample rate is typically not limited in any way as is the case for the O_2_ and CO_2_ measurements, these findings imply that PA should preferrably be sampled at a higher rate than the respiratory exchange.

We evaluated the effect of the chamber size and the flow rate on the performance of the P-spline model by simulating data for various washout times 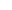. For metabolic chamber systems for small rodents 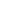 typically lies in the range of several minutes. For instance, U.S. guidelines suggest a minimal cage size of 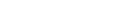 for mice [24], which results in a value of 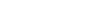 when the flow rate is set to 0.4 l/min. In contrast, for larger metabolic chambers, such as those employed for large mammals and humans, 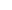 can amount up to several hours [25]. From the simulation study it followed that when 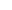 was increased from 15 to 45 minutes, the estimation accuracy decreased considerably. This suggests that for these values of 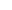 the fast time variations in the TEE that are due to activity are filtered out. As a result, the time-dependent AEE and RMR both approximate a sine-shape, which makes it difficult to separate them by means of regression.

Interestingly, the simulation study also predicted that more accurate estimates of RMR could be obtained for shorter sample times 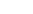, irrespective of the chamber size. This finding was surprising at first sight, since from Nyquist’s sampling theorem it follows that there is no point in sampling low pass filtered (i.e. bandlimited) signals at a high rate [26]. However, since measurement noise is present on the TEE, short sample times have the function of noise attenuation and hence improve the estimation accuracy.

### Comparison of Estimation Methods

We compared the performance of the P-spline regression model with that of conventional methods for estimating AEE and RMR. It was found that the errors made by linear regression and the TEE averaging for zero activity method were considerably larger than that of the P-spline model, both in estimating average and time-dependent RMR. The large error in the time-dependent RMR estimate could be explained by the fact that these methods did not take the time variations in the RMR into account. In contrast, the inaccuracies in the average RMR were caused by the bias in these approaches, which can be attributed to the diurnal correlation between RMR and PA (i.e. mice are more active and have a higher RMR during the night) and to the non-negligible measurement error in the PA.

In contrast, a higher overall accuracy was obtained by the Kalman filter. The estimation error of the average RMR was comparable to that of the P-spline method for sample times under 10 min, while it was slightly larger for higher 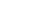. However, the estimation error of the time-dependent RMR of the Kalman filter was considerably larger than that of the P-spline method for both high and low sample rates. A possible explanation for this is that the Kalman filter is less able to cope with activity data that has been measured with infrared beam sensors, because of the high degree of noise on this data. Another explanation could be that the Kalman filter corresponds to a causal filter and bases its predictions on only a few past measurements, which means that there is a relatively large uncertainty in the RMR estimate for each time point. In contrast, by its design the P-spline model bases its estimates on a larger set of local data points, making it therefore more robust under both frequent and infrequent sampling regimes.

We also observed that the estimation error of the Kalman filter increased more rapidly with the sample time. This larger sensitivity to low sample rates is probably due to the fact that the Kalman filter requires the activity and TEE data to be measured with the same sample rate, which means that it cannot properly exploit high time resolution activity measurements. In fact, as we argued before, increasing the sample time of the activity measurements decreases its correlation with the energy expenditure and therefore increases the estimation error of the RMR (Fig. 3A–C).

Finally, a difficulty that we encountered with the use of the Kalman filter is that the filter parameters (i.e. the process noise variances) needed to be determined *a priori* by the user. In the simulation study we fitted these parameters for each sample rate as to minimise the estimation error, such that we could compare the P-spline regression model with the optimal performance of the Kalman filter. In practice, however, one may not be able to attain the same accuracy with the Kalman filter on real data, because no criteria exist for automatic selection of the filter parameters and manual selection is difficult [14].

### Analysis of Experimental Data

We illustrated the value of our method in a case study where we calculated AEE and RMR in male and female C57Bl/6J mice that had been put on a 10 week high fat diet. Since mice were housed below their thermoneutral zone, the estimated RMR also included energy expenditure for thermoregulation [9]. We found that TEE in male mice was higher due to a difference in RMR and that even though female mice were more active, the lower caloric cost of activity in female mice caused AEE levels to be similar in both groups. The most probable cause of this effect was the difference in body weight, since heavier mice are known to be less prone to physical activity [27], but also need more energy to move. This study demonstrates the importance of including TEE decomposition in the analysis of indirect calorimetry data, as it permits to explain observed differences in TEE and to determine the effect of PA on energy expenditure.

Interestingly, we found a strongly nonlinear relationship between the number of infrared beam breaks and the energy expenditure. There are several possible explanations for this phenomenon. First of all the nonlinearity could be an inherent property of the activity sensor. For instance, activity bouts that are characterised by typical non-displacement types of behaviour as grooming or eating may yield relatively few beam breaks per expended energy while displacement types of behaviour as foraging or exploring can yield more beam breaks. Another explanation could be that since under room temperature a considerable part of RMR in mice is due to nonshivering thermogenesis, this energy component is temporarily downregulated during periods of activity because enough waste heat is produced due to activity. Both hypotheses will need to be investigated further in future studies in which other activity sensors are used and the ambient temperature is changed.

From the data generated in the case study we could not conclude that the lower level of activity in male mice had also played a causal role in their increased weight gain, as has been reported elsewhere [6]. In fact, from short indirect calorimetry experiments it is difficult to explain weight gain in terms of adaptations in RMR or PA, since both RMR and PA correlate with body weight [27], [28]. We therefore envisage that applying the P-spline model to longitudinal indirect calorimetry datasets (i.e. for a period of several weeks) will permit to estimate the gradual changes in RMR and AEE and will provide a better insight into the causal factors of the development of overweight in rodents.

In addition, estimation of the time-dependent RMR enables a detailed analysis of energy metabolism. For instance, the RMR time sequence can be used to quantify the presence of circadian rhythm, which permits to investigate the effect of genetic [29] and dietary factors [30] on circadian rhythm in energy expenditure. Furthermore, the response in RMR due to experimental interventions can be determined, which permits to measure the effect of caloric restriction on energy expenditure [31], [32] and to quantify the thermic effect of food as the increase in RMR following a feeding bout [16], [33], [34]. Clearly, in order to be able to assess transient effects in the RMR with a reasonable amount of precision, it is important that both activity and respiratory exchange are measured with a high enough sample rate and that a high knot number is chosen.

A possible extension of our method is to incorporate activity data from different sensors. If a metabolic chamber is equipped with both infrared beam sensors and a running wheel, the number of wheel revolutions can be included in the P-spline regression model as an additional activity signal. In this way, a distinction can be made between AEE from spontaneous physical activity (SPA), i.e. non-exercise activity thermogenesis (NEAT), and AEE from voluntary exercise [35]–[37]. Future research will need to establish how the delay in oxygen consumption due to anaerobic energy expenditure and excess-post exercise oxygen consumption can be modelled reliably such that accurate estimates of exercise related energy expenditure can be obtained.

In conclusion, we have developed a method for estimating AEE and RMR from activity and indirect calorimetry data that is based on regression with penalised splines. Our method gives robust results even in cases when activity measurements are noisy and respiratory exchange is sampled at a low rate. We validated the P-spline estimation method extensively on simulated datasets and illustrated the value of TEE decomposition in a case study involving high fat diet fed male and female C57Bl/6J mice. Since we have developed our method based on a general mathematical model that involves the time variation in RMR, the effect of gas diffusion on the measured TEE and the measurement error in TEE and PA, we are confident that its applicability extends to other indirect calorimetry systems.

## Methods

### Ethics Statement

The institutional Ethical Committee on Animal Care and Experimentation from Leiden University Medical Center has approved all experiments under permit number 07007 (high fat diet case study) and 10093 (high time resolution metabolic chamber experiment).

### Penalised Spline Regression Model

#### Problem statement

We first provide a formalisation of the decomposition of total energy expenditure that is based on estimation theory. Letting 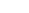 be a vector of dimension *n*×1 that contains the time instants at which TEE has been sampled, we formulate the decomposition of TEE into RMR and AEE as 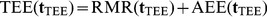
(1)where 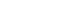, 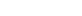 and 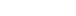 are *n*×1 vectors that represent the time series of TEE, RMR and AEE respectively, at sample times 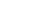. Obtaining estimates of the terms on the right-hand side of (1) is based on the additional knowledge that is available of the AEE, namely that its variation in time correlates with the measured physical activity. In the specific case that a linear relationship exists between PA and AEE, we have
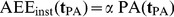
(2)where 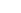 is an 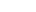 vector that contains the time instants at which PA has been sampled and 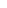 the caloric cost of activity (CCA), i.e. the conversion factor between measured activity and the expended energy related to this activity. In practice, however, the relation between PA and AEE may be nonlinear, in which case the raw PA measurements need to be preprocessed by an appropriately chosen function such that Eq. (2) holds; see Supplementary Text S2 for details.

Since activity measurements are instantaneous, (2) is an expression of the activity related energy expenditure as it would be measured at the level of the cell (indicated by the subscript *inst*). In contrast, the respiratory exchange of the subject as it is actually picked up by the gas sensors has been distorted by diffusion effects [14], [19], [20], [38]. Assuming that the gas diffusion can be modelled by two linear compartments, representing the subject and the chamber, the signal deformation is characterised by the transfer function
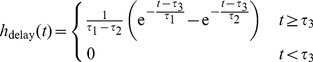
(3)where 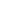 is the washout time introduced by the subject, which mainly depends on the amounts of O_2_ and CO_2_ that are dissolved in the blood, 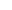 is the washout time of the chamber, which equals the ratio of the chamber volume to the air flow through it, and 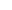 the time delay that is introduced by the tubing and gas dryers that are located between the chamber and gas sensors. It is important to note that for the purpose of TEE decomposition, 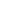 may not be too large because otherwise the high frequency variations in TEE that result from activity are filtered out.

Having an expression for 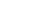, the activity related energy expenditure as it would be measured at the level of the gas sensors can be inferred by convolving 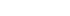 with 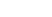. Approximating the continuous time convolution by summing over the discrete sample times 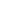, we obtain the matrix multiplication
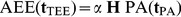
(4)with **H** the *n*×*m* matrix with elements 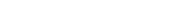, where square brackets denote the position within a vector.

Inserting (4) into (1) gives a general formulation of TEE decomposition as an estimation problem
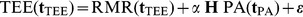
(5)The term **e** refers to the random errors that are due to measurement errors in TEE and to the approximation made by modelling AEE as a function of PA. Estimation methods of AEE and RMR from the TEE and PA time series can be seen as specific approaches to solve (5).

#### Penalised spline regression

The regression model that we propose for TEE decomposition is based on the assumption that the time variation in the RMR can be modelled by means of cubic spline functions
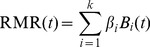
(6)with 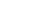 the cubic B-spline basis functions with coefficients 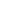 and with knots at equidistant locations. The number of knots  has to be chosen *a priori* based on the frequency components in RMR that one wants to estimate (see Results). Inserting (6) into (5), we obtain the model
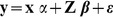
(7)with 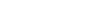 the measured TEE time sequence, 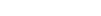 the time sequence of the intensity of activity that accounts for gas diffusion effects, Z the n×k design matrix containing the spline basis functions evaluated at the sample times 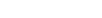, 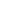 the cost of activity and 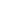 the k×1 vector of spline coefficients.

Assuming the error vector **e** to be normal, zero mean and independent and identically distributed 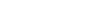, with **I** the identity matrix, 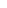 and 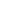 can be estimated from the penalised least squares criterion
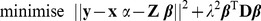
(8)with 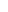 the smoothing parameter and 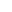 the 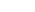 penalisation matrix. The second term in (8) corresponds to the roughness penalty that is used in nonparametric regression to reduce the variability of the spline coefficient estimates [17], [22], [39]; hence, (7) corresponds to a penalised spline regression model. We here propose to penalise the square of the first derivative of the RMR time series; from (6) it then follows that 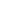 is defined as 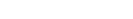. Solving (8) for 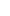 gives the estimate 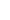 of the spline coefficients
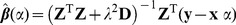
(9)Since in practice the measured level of physical activity is not an exact predictor of the AEE, the term 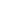 in (7) is only known approximately. From regression theory it is known that when there is uncertainty in the predictor variables, the conventional least squares approach (8) will yield biased results [40]. As it is evident that the uncertainty in 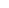 is larger during periods of activity than during inactivity, we propose a multiplicative error model
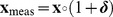
(10)with 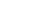 the 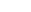 vector containing the measured activity, 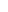 the measurement error that reflects the uncertainty in 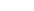 and 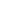 the entry-wise (Hadamard) vector product. In practice 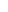 reflects inaccuracies in the measurement of PA and variations in the cost of activity.

Following the approach of Nakamura and Zhong *et al.* [41], [42], a corrected least squares function for a multiplicative errors-in-variables model can be constructed, yielding an unbiased estimate of 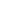


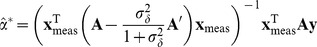
(11)with 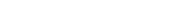 and 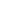 the 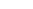 matrix that contains the diagonal elements of 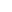 and zeros otherwise. See Supplementary Text S1 for a derivation of (11).

The estimates 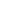 and 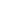 depend on the smoothing parameter 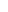 and on the measurement error variance 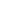. Since generally these parameters are unknown, values need to be derived from the data. For nonparametric regression models the optimal degree of smoothing 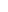 is typically found by maximising some appropriately chosen measure of goodness of fit, such as Akaike’s Information Criterion [22], [43], Generalised Cross Validation [18], [44] or maximum likelihood [17]; in this work we have used the Generalised Cross Validation criterion. An estimate of 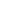 can be obtained from the model’s residuals 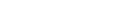. As the residuals vary heteroskedastically depending on the size of the measurement error variance, it is possible to construct a likelihood function that permits to estimate 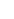. See Supplementary Text S1 for details.

### Data Acquisition

#### Simulated indirect calorimetry data

Energy expenditure and physical activity datasets were simulated attempting to resemble experimental indirect calorimetry data from mice as closely as possible. In short, a total of 500 datasets of a length of 3 days were simulated, consisting each of a TEE, AEE, RMR and PA time series that were sampled with a time interval of 10 s. Activity patterns were simulated as a sequence of activity bouts of varying intensity and duration, which served to derive the AEE and PA time sequence. The AEE was calculated from the activity pattern by multiplying it with the cost of activity, which was varied between activity bouts. The PA was calculated by scaling the activity pattern function to a probability function and by subsequently randomly drawing infrared beam breaks, which were then binned into 10 s time intervals. The RMR time sequence was modelled as a cosine function to mimic the typical day-night variation and a Gaussian stochastic process filtered by a low pass filter to simulate additional slow time variations. The average RMR was assumed to be 10 kcal/day for each of the 500 datasets. The TEE was calculated as the added AEE and RMR time series, plus measurement noise. See Supplementary Text S4 for details.

#### High time resolution indirect calorimetry data

Energy metabolism of a single male C57Bl/6J mouse (age 12 weeks; chow diet) was measured by means of indirect calorimetry. The mouse was housed at a temperature of 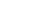 and subjected to individual indirect calorimetry measurements for a period of 4 consecutive days under a 12h light-dark cycle (07:00–19:00) (Comprehensive Laboratory Animal Monitoring System, CLAMS; Columbus Instruments). The first 24 hours of the experiment served to allow acclimatisation of the animal to the cage and were not included in the analysis. Food and water were available *ad libitum* during the whole experiment, and intake was analysed every 10 seconds. Spontaneous PA was measured as infrared beam breaks in X and Z direction every 10 seconds. The interbeam space was 12 mm in X direction and 38 mm in Z direction. Total PA was taken as the total of counts in X and Z direction divided by the sample time expressed in minutes. Oxygen consumption and carbon dioxide production rate measurements were performed at intervals of 10 seconds throughout the whole period. A single measurement of the external air was taken at the start of the experiment to serve as reference. Total energy expenditure was calculated according to Lusk [45]. During the experiment situations were avoided that could interfere with the TEE and PA measurements and compromise their time correlation. It was ascertained that no infrared beams were occluded and that the cage was not opened more than once a day for replenishment of the feeder.

#### Case study

Energy metabolism of 8 male and 7 female C57Bl/6J mice (age 24 weeks) that had been put on a 10 week high fat diet (D12451, Research Diet Services) was measured in the indirect calorimeter as stated above, with the exception that PA was measured at 60 second intervals and 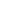 consumption, 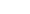 production and reference air concentrations were measured every 7.5 minutes.

### Data Analysis

The simulated data served to determine the accuracy of the P-spline method in estimating the average and time-dependent RMR. The estimation error was calculated as the Root Mean Square Error (RMSE) and reported as either the absolute RMSE or the relative RMSE, i.e. the RMSE divided by the average RMR which was 10 kcal/day for all simulated datasets.

#### Knot number selection

Influence of the knot number 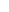 on the performance of the P-spline method was determined by estimating time-dependent RMR with 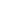 ranging between 1 and 100 knots/day. For each estimated RMR time sequence, the error time sequence was calculated by subtracting the actual RMR. Since the fast time variations in the TEE were due to both RMR and AEE, the high frequency components in RMR were more difficult to estimate. Therefore, we calculated the RMSE for 5 different frequency ranges: for 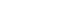, 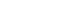, 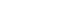, 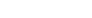 and for all frequencies 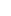. In detail, for a given cutoff frequency 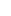 the discrete Fourier Transform of the error time sequence was calculated and frequency components above 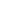 were removed [46]. Subsequently, the inverse Fourier Transform was calculated and the RMSE was determined. Note that the first frequency range corresponds to estimating the average RMR. Since the high frequency components in the RMR could not be estimated accurately because of interference of the measurement error in PA and TEE, for the remainder of the validation study it was chosen to evaluate the performance of the time-dependent RMR estimation by focusing on frequency components below 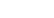. The knot number of the P-spline method was set to 15 knots/day.

#### Validation of the P-spline model

Influence of the sample rate of TEE and PA on the accuracy of the P-spline model was determined by calculating the RMSE of the average and time-dependent RMR first in the situation where 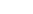 and 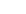 were equal and ranged between 10 s and 20 min and second in the situation where 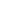 was set to 10 s and 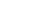 ranged between 10 s and 20 min. In the first situation also the standard deviation in the residuals of the P-spline model was calculated, such that it could be determined whether the unexplained variance increased with 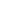 and whether this was similar for the experimental high time resolution dataset. Lower sample rates of PA were emulated by taking every 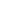-th data point and adding the previous 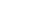 data points to it since beam breaks were reported cumulatively by the CLAMS metabolic chamber system.

The effect of 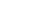 on the estimation accuracy was assessed by calculating the downsampling induced variability (DIV) for simulated and experimental data. In short, lower sample rates of TEE were emulated by taking every 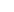-th data point in the time series data, which was carried out 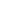 different times creating 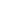 separate downsampled datasets from the original data. The variation between the 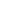 distinct estimates was taken as a measure of the estimation error that was introduced by a reduction in sample resolution. See Supplementary Text S5 for details.

Influence of the chamber size and flow rate on the estimation accuracy was determined by simulating datasets for different washout times 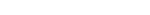 and subsequently by calculating the RMSE for the average and time-dependent RMR estimate.

#### Performance comparison with other methods

The performance of the P-spline model was compared with that of three other TEE decomposition methods: linear regression, zero activity TEE averaging, and Kalman filtering (Supplementary Text S3). The performance was evaluated as the accuracy in estimating average and time-dependent RMR for 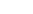 ranging between 10 s and 20 min. Bias in the average RMR estimate of each method was determined by calculating the mean error in average RMR for all 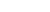 datasets.

#### Experimental data

The high time resolution experimental data served to determine whether the measured PA required preprocessing for our metabolic chamber system and how this function should be parameterised. Also, the data was used to infer the parameters with which the time-dependent RMR was generated in the simulation study. See Supplementary Text S2 and S4 for details.

From the experimental data generated for the case study the average RMR, AEE, CCA and time-dependent RMR and AEE were determined separately for each mouse by means of the P-spline model. Time-dependent estimates of RMR were used to determine the presence of circadian rhythm by fitting a cosine function of a 24 h period, after linear and quadratic trends had been removed. Body weight, CCA, circadian amplitude in the RMR and average TEE, RMR, AEE, PA were compared between male and female mice with a two-tailed *t*-test assuming unequal variances. Correlations of the metabolic parameters with body weight were tested with a one-way analysis of variance. 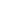-values less than 0.05 were considered statistically significant.

The validation study and statistical analyses were performed in MATLAB (The MathWorks). The P-spline regression model and other TEE decomposition algorithms were also implemented in MATLAB. Details regarding the calculations performed for each method can be found in the Supplementary Text S1 and S3. The developed MATLAB functions for performing TEE decomposition – in particular, the functions for fitting the P-spline regression model and the activity preprocessing parameters – are available upon request.

## Supporting Information

Text S1 Technical details of the penalised spline regression model.
(PDF)

Text S2 Characterisation of the relation between the measured physical activity and related energy expenditure.
(PDF)

Text S3 Detailed discussion of the conventional methods for estimating AEE and RMR: linear regression, averaging TEE for periods of zero activity and Kalman filtering.
(PDF)

Text S4 Simulation protocol of indirect calorimetry data.
(PDF)

Text S5 Calculation of the downsampling induced variability.
(PDF)

## References

[1] Ravussin E, Lillioja S, Anderson TE, Christin L, Bogardus C (1986). Determinants of 24-hour energy expenditure in man – methods and results using a respiratory chamber.. J Clin Invest.

[2] Dauncey MJ (1990). Activity and energy expenditure.. Can J Physiol Pharmacol.

[3] Levine JA, Eberhardt NL, Jensen MD (1999). Role of nonexercise activity thermogenesis in resistance to fat gain in humans.. Science.

[4] Weinsier RL, Hunter GR, Desmond RA, Byrne NM, Zuckerman PA (2002). Free-living activity energy expenditure in women successful and unsuccessful at maintaining a normal body weight.. Am J Clin Nutr.

[5] Novak CM, Kotz CM, Levine JA (2006). Central orexin sensitivity, physical activity, and obesity in diet-induced obese and diet-resistant rats.. Am J Physiol Endocrinol Metab.

[6] Bjursell M, Gerdin AK, Lelliott CJ, Egecioglu E, Elmgren A (2008). Acutely reduced locomotor activity is a major contributor to Western diet-induced obesity in mice.. Am J Physiol Endocrinol Metab.

[7] Blaxter K (1989). Energy Metabolism in Animals and Man..

[8] Bursztein S, Elwyn DH, Askanazi J, Kinney JM (1989). Energy metabolism, indirect calorimetry, and nutrition..

[9] B C, Nedergaard J (2011). Nonshivering thermogenesis and its adequate measurement in metabolic studies.. J Exp Biol.

[10] Ravussin E, Lillioja S, Knowler WC, Christin L, Freymond D (1988). Reduced rate of energy expenditure as a risk factor for body-weight gain.. N Eng J Med.

[11] Tataranni PA, Harper IT, Snitker S, Del PA, Vozarova B (2003). Body weight gain in freeliving Pima Indians: effect of energy intake vs expenditure.. Int J Obes Rel Metab Disord.

[12] MacLean P, Higgins JA, Johnson GC, Fleming-Elder BK, Donahoo WT (2004). Enhanced metabolic efficiency contributes to weight regain after weight loss in obesity-prone rats.. Am J Physiol Regul Integr Comp Physiol.

[13] Nonogaki K, Abdallah L, Goulding EH, Bonasera SJ, Tecott LH (2003). Hyperactivity and reduced energy cost of physical activity in serotonin 5-HT(2C) receptor mutant mice.. Diabetes.

[14] Even PC, Perrier E, Aucouturier JL, Nicolaidis S (1991). Utilisation of the method of Kalman filtering for performing the on-line computation of background metabolism in the free-moving, free-feeding rat.. Physiol Behav.

[15] Deveaux V, Cadoudal T, Ichigotani Y, Teixeira-Clerc F, Louvet A (2009). Cannabinoid CB2 receptor potentiates obesity-associated inflammation, insulin resistance and hepatic steatosis.. PLoS One.

[16] Stepien M, Gaudichon C, Azzout-Marniche D, Fromentin G, Tomé D (2010). Postprandial nutrient partitioning but not energy expenditure is modified in growing rats during adaptation to a high-protein diet.. J Nutr.

[17] Ruppert D, Wand MP, Carroll RJ (2003). Semiparametric Regression..

[18] Craven P, Wahba G (1979). Smoothing noisy data with spline functions: Estimating the correct degree of smoothing by the method of generalized cross-validation.. Numer Math.

[19] Arch JR, Hislop D, Wang SJ, Speakman JR (2006). Some mathematical and technical issues in the measurement and interpretation of open-circuit indirect calorimetry in small animals.. Int J Obes (Lond).

[20] Lighton JRB (2008). Measuring metabolic rates: a manual for scientists..

[21] Tschöp MH, Speakman JR, Arch JR, Auwerx J, Brüning JC (2011). A guide to analysis of mouse energy metabolism.. Nat Methods.

[22] Eilers PHC, Marx BD (1996). Flexible smoothing with B-splines and penalties.. Statist Sc.

[23] Cooper CE, Withers PC (2010). Effect of sampling regime on estimation of basal metabolic rate and standard evaporative water loss using flow-through respirometry.. Physiol Biochem Zool.

[24] Institute for Laboratory Animal Research (2011). Guide for the Care and Use of Laboratory Animals..

[25] Lighton JRB, Halsey LG (2010). Flow-Through respirometry applied to chamber systems: Pros and cons, hints and tips.. Comp Biochem Physiol A Mol Integr Physiol.

[26] Proakis J, Manolakis D (1992). Digital Signal Processing: Principles, Algorithms, and Applications..

[27] Tou JCL, Wade CE (2002). Determinants affecting physical activity levels in animal models.. Exp Biol Med.

[28] Kaiyala KJ, Morton GJ, Leroux BG, Ogimoto K, Wisse B (2010). Identification of body fat mass as a major determinant of metabolic rate in mice.. Diabetes.

[29] Liu C, Li S, Liu T, Borjigin J, Lin JD (2007). Transcriptional coactivator pgc-1alpha integrates the mammalian clock and energy metabolism.. Nature.

[30] Kohsaka A, Laposky AD, Ramsey KM, Estrada C, Joshu C (2007). High-fat diet disrupts behavioral and molecular circadian rhythms in mice.. Cell Metab.

[31] Dulloo AG, Girardier L (1990). Adaptive changes in energy expenditure during refeeding following low-calorie intake: evidence for a specific metabolic component favoring fat storage.. Am J Clin Nutr.

[32] Even PC, Nicolaidis S (1993). Adaptive changes in energy expenditure during mild and severe feed restriction in the rat.. Br J Nutr.

[33] Even PC, Mokhtarian A, Pele A (1994). Practical aspects of indirect calorimetry in laboratory animals.. Neurosci Biobehav Rev.

[34] van Milgen J, Noblet J, Dubois S, Bernier JF (1997). Dynamic aspects of oxygen consumption and carbon dioxide production in swine.. Br J Nutr.

[35] Levine JA (2002). Non-exercise activity thermogenesis (NEAT).. Best Pract Res Clin Endocrinol Metab.

[36] Garland TJ, Schutz H, Chappell MA, Keeney BK, Meek TH (2011). The biological control of voluntary exercise, spontaneous physical activity and daily energy expenditure in relation to obesity: human and rodent perspectives.. J Exp Biol.

[37] Morton GJ, Kaiyala KJ, Fisher JD, Ogimoto K, Schwartz MW (2011). Identification of a physiological role for leptin in the regulation of ambulatory activity and wheel running in mice.. Am J Physiol Endocrinol Metab.

[38] Tokuyama K, Ogata H, Katayose Y, Satoh M (2009). Algorithm for transient response of whole body indirect calorimeter: deconvolution with a regularization parameter.. Appl Physiol.

[39] Green PJ (1987). Penalized likelihood for general semi-parametric regression models.. Int Statist Rev.

[40] Fuller WA (1987). Measurement Error Models..

[41] Nakamura T (1990). Corrected score function for errors-in-variables models: Methodology and application to generalized linear models.. Biometrika.

[42] Zhong XP, Fung WK, Wei BC (2002). Estimation in linear models with random effects and errorsin-variables.. Ann Inst Statist Math.

[43] Akaike H (1974). A new look at the statistical model identification.. IEEE Trans Automat Control.

[44] Ruppert D (2002). Selecting the number of knots for penalized splines.. J Comp Graph Statist.

[45] Lusk G (1928). The elements of the science of nutrition..

[46] Oppenheim AV, Schafer RW, Buck JR (1999). Discrete-time signal processing..

