# Supplementary material for: Estimation of Activity Related Energy Expenditure and Resting Metabolic Rate in Freely Moving Mice from Indirect Calorimetry Data
Source: PLoS One. 2012 May 4;7(5):e36162. doi: 10.1371/journal.pone.0036162 (PMC3344840; doi:10.1371/journal.pone.0036162)
Supplement: Text S1 — Technical details of the penalised spline regression model. (PDF) [file pone.0036162.s001.pdf]

Supplemental Material Text S1

Estimation of Activity Related Energy Expenditure and Resting Metabolic Rate in Freely Moving Mice from Indirect Calorimetry Data

Jan Bert van Klinken[1], Sjoerd A.A. van den Berg, Louis M. Havekes, Ko Willems van Dijk

Penalised spline regression model

Unbiased estimation of caloric cost of activity. The penalised spline regression model with multiplicative errors-in-variables is defined as

$$\mathbf{y} = \mathbf{x}\,\alpha + \mathbf{Z}\,\boldsymbol{\beta} + \boldsymbol{\varepsilon} \tag{1}$$

$$\mathbf{x}_{\text{meas}} = \mathbf{x} \circ (1 + \boldsymbol{\delta}) \tag{2}$$

with $\mathbf{y} = \text{TEE}(\mathbf{t}_{\text{TEE}})$ the measured TEE time sequence, $\mathbf{x}$ the time sequence with the intensity of PA, $\mathbf{x}_{\text{meas}}$ the measured intensity of PA, $\mathbf{Z}$ the $n \times k$ design matrix containing the spline basis functions evaluated at the sample times $z_{ji} = B_i(\mathbf{t}_{\text{TEE}}[j])$, α the cost of activity and $\boldsymbol{\beta}$ the $k \times 1$ vector of spline coefficients. The error term $\boldsymbol{\varepsilon}$ is assumed to be normal, zero mean and independent and identically distributed $\boldsymbol{\varepsilon} \sim \mathcal{N}(\mathbf{0}, \sigma_\varepsilon^2 \mathbf{I})$.

Let SS be the the penalised residual sum of squares function that is minimised to estimate α and $\boldsymbol{\beta}$

$$SS(\alpha, \boldsymbol{\beta}) = ||\mathbf{y} - \mathbf{x}\,\alpha - \mathbf{Z}\,\boldsymbol{\beta}||^2 + \lambda^2 \boldsymbol{\beta}^{\mathrm{T}} \mathbf{D} \boldsymbol{\beta} \tag{3}$$

with λ the smoothing parameter and $\mathbf{D}$ the $k \times k$ penalisation matrix. Minimising (3) for $\boldsymbol{\beta}$ gives the estimate $\hat{\boldsymbol{\beta}}$ of the spline coefficients

$$\hat{\boldsymbol{\beta}}(\alpha) = \left(\mathbf{Z}^{\mathrm{T}}\mathbf{Z} + \lambda^2 \mathbf{D}\right)^{-1} \mathbf{Z}^{\mathrm{T}}(\mathbf{y} - \mathbf{x}\,\alpha) \tag{4}$$

which is unbiased in the case $\mathbf{x}$ is corrupted with measurement error, meaning that $\mathbf{x}$ in (4) may be replaced by $\mathbf{x}_{\text{meas}}$. Inserting (4) into (3) gives the sum of squares function for estimating α in the case of zero measurement error

$$SS'(\alpha) = (\mathbf{y} - \mathbf{x}\,\alpha)^{\mathrm{T}} \mathbf{A} (\mathbf{y} - \mathbf{x}\,\alpha) \tag{5}$$

[1]E-mail: J.B.van_Klinken@lumc.nl

with $\mathbf{A} = \mathbf{I} - \mathbf{Z}(\mathbf{Z}^{\mathrm{T}}\mathbf{Z} + \lambda^2\mathbf{D})^{-1}\mathbf{Z}^{\mathrm{T}}$. In the case measurement error is present, (5) will give a biased estimate of α if $\mathbf{x}$ is replaced by $\mathbf{x}_{\mathrm{meas}}$ because of regression dilution [1]. We now propose a corrected sums of squares function $SS^*(\alpha)$ that gives an unbiased estimate of α, following the approach of Nakamura and Zhong *et al.* [2,3]. In order for $SS^*(\alpha)$ to be an unbiased estimator of α, it must satisfy

$$\begin{aligned} E^*\left(\frac{d\,SS^*(\alpha)}{d\,\alpha}\right) &= \frac{d\,SS'(\alpha)}{d\,\alpha} \\ &= 2\,\mathbf{x}^{\mathrm{T}}\mathbf{A}\mathbf{x} - 2\,\mathbf{x}^{\mathrm{T}}\mathbf{A}\mathbf{y}\alpha \end{aligned}$$

where E^* is the expectation with respect to $\mathbf{x}_{\mathrm{meas}}$, given $\mathbf{y}$ and $\boldsymbol{\beta}$. Assuming a distribution of the PA measurement error $\boldsymbol{\delta} \sim \mathcal{N}(\mathbf{0}, \sigma_\delta^2\mathbf{I})$, it follows from (2) that the measured activity time series has a distribution $\mathbf{x}_{\mathrm{meas}} \sim \mathcal{N}(\mathbf{x}, \sigma_\delta^2\mathbf{D}_{\mathbf{x}}^2)$, with $\mathbf{D}_{\mathbf{x}}$ the diagonal matrix with the elements of $\mathbf{x}$ on the diagonal. Therefore, it holds that

$$\begin{aligned} E^*\left(\mathbf{x}_{\mathrm{meas}}^{\mathrm{T}}\mathbf{A}\mathbf{x}_{\mathrm{meas}}\right) &= \mathbf{x}^{\mathrm{T}}\mathbf{A}\mathbf{x} + \sigma_\delta^2\ \mathrm{trace}\left(\mathbf{A}\ \mathbf{D}_{\mathbf{x}}^2\right) \\ &= \mathbf{x}^{\mathrm{T}}\left(\mathbf{A} + \sigma_\delta^2\ \mathbf{A}'\right)\mathbf{x} \end{aligned} \tag{6}$$

with $\mathbf{A}'$ the matrix that contains the diagonal elements of $\mathbf{A}$ and zeros otherwise. Given (6), it is readily shown that the condition for unbiasedness is satisfied by the following least squares criterion

$$SS^*(\alpha) = (\mathbf{y} - \mathbf{x}_{\mathrm{meas}}\,\alpha)^{\mathrm{T}}\mathbf{A}(\mathbf{y} - \mathbf{x}_{\mathrm{meas}}\,\alpha) - \frac{\sigma_\delta^2}{1+\sigma_\delta^2}\mathbf{x}_{\mathrm{meas}}^{\mathrm{T}}\mathbf{A}'\mathbf{x}_{\mathrm{meas}}\alpha^2 \tag{7}$$

Minimising SS^* for α then gives an unbiased estimate of the caloric cost of activity for the multiplicative errors-in-variables model (1) and (2)

$$\hat{\alpha}^* = \left(\mathbf{x}_{\mathrm{meas}}^{\mathrm{T}}\left(\mathbf{A} - \frac{\sigma_\delta^2}{1+\sigma_\delta^2}\mathbf{A}'\right)\mathbf{x}_{\mathrm{meas}}\right)^{-1}\mathbf{x}_{\mathrm{meas}}^{\mathrm{T}}\mathbf{A}\mathbf{y} \tag{8}$$

Information criteria for determining λ and σ_δ^2. The estimates $\hat{\alpha}$ and $\hat{\boldsymbol{\beta}}$ depend on the smoothing parameter λ and on the measurement error variance σ_δ^2. Since generally these parameters are unknown, values need to be derived from the data. For nonparametric regression models the optimal degree of smoothing λ is typically found by maximising some appropriately chosen measure of goodness of fit, such as Akaike's Information Criterion [4,5], Generalised Cross Validation [6,7] or maximum likelihood [8]. We found that in our case the Generalised Cross Validation (GCV) criterion gives good results

$$\mathrm{GCV} = \frac{|\mathbf{y} - \mathbf{x}_{\mathrm{meas}}\hat{\alpha} - \mathbf{Z}\hat{\boldsymbol{\beta}}|^2}{\left(1 - \frac{1}{n'}\,df\right)^2} \tag{9}$$

with *df* the degrees of freedom, defined as $df = \text{trace}\left((\mathbf{Z}^{\mathrm{T}}\mathbf{Z} + \lambda^2\mathbf{D})^{-1}\mathbf{Z}^{\mathrm{T}}\mathbf{Z}\right)$ [8]. Instead of the ordinary definition of the GCV where the degrees of freedom are divided by the number of datapoints $n = \frac{t_{\text{duration}}}{T_{\text{TEE}}}$ (t_{duration} the time duration of the experiment), we used a definition that was independent of the sample time: $n' = \frac{t_{\text{duration}}}{T'_{\text{TEE}}}$, with T'_{TEE} set to a fixed value of 10 min. This adjustment served to make the degree of smoothing λ that results from minimising (9) insensitive to the sample rate, which ensured that no overfitting occurred for high sample rates. The value of $T'_{\text{TEE}} = 10$ min was chosen since the traditional GCV was found to give good results for that sample time. Note that since the prediction error $|\mathbf{y} - \mathbf{x}_{\text{meas}}\hat{\alpha} - \mathbf{Z}\hat{\boldsymbol{\beta}}|^2$ does not change much when the biased estimate $\hat{\alpha}$ is used instead of the unbiased estimate $\hat{\alpha}^*$, the GCV is practically independent of σ_δ^2; hence, we chose to select the smoothing parameter λ assuming zero measurement error $\sigma_\delta^2 = 0$, which reduced computational demands.

Estimating the variance of the measurement error σ_δ^2 from the data is more complicated. To our knowledge, no unbiased estimator of σ_δ^2 currently exists that applies to our setting. Nevertheless, an approximation of σ_δ^2 can be obtained by quantifying the degree of heteroscedasticity with which the residuals of the P-spline model vary, since this variation contains information about the size of σ_δ^2. We have for the residuals

$$
\begin{aligned}
\mathbf{e} &= \mathbf{y} - \mathbf{x}_{\text{meas}}\hat{\alpha}^* - \mathbf{Z}\hat{\boldsymbol{\beta}} \\
&= \mathbf{x}(\alpha - \hat{\alpha}^*) + \mathbf{Z}(\boldsymbol{\beta} - \hat{\boldsymbol{\beta}}) + \boldsymbol{\varepsilon} + \hat{\alpha}^*\mathbf{x} \circ \boldsymbol{\delta} \qquad (10)
\end{aligned}
$$

Neglecting the variance in the estimates $\hat{\alpha}^*$ and $\hat{\boldsymbol{\beta}}$, the residuals follow a distribution $\mathbf{e} \sim \mathcal{N}(\mathbf{0}, \sigma_\varepsilon^2\mathbf{I} + \sigma_\delta^2\hat{\alpha}^{*2}\mathbf{D}_{\mathbf{x}}^2)$; that is, the variance is heteroscedastic, depending on $\mathbf{x}$, $\hat{\alpha}^*$ and σ_δ^2. This allowed us to construct a likelihood function for estimating σ_δ^2 and σ_ε^2

$$
\begin{aligned}
\ell &= -\frac{1}{2}\left[\log\det \boldsymbol{\Sigma}_e + \mathbf{e}^{\mathrm{T}}\boldsymbol{\Sigma}_e^{-1}\mathbf{e}\right] \\
&= -\frac{1}{2}\left[\log\det \boldsymbol{\Sigma}_e + (\mathbf{y} - \mathbf{x}_{\text{meas}}\hat{\alpha}^*)^{\mathrm{T}}\,\mathbf{A}\boldsymbol{\Sigma}_e^{-1}\mathbf{A}\,(\mathbf{y} - \mathbf{x}_{\text{meas}}\hat{\alpha}^*)\right] \qquad (11)
\end{aligned}
$$

with $\boldsymbol{\Sigma}_e$ the covariance matrix of the residuals, which can be approximated by $\boldsymbol{\Sigma}'_e = \sigma_\varepsilon^2\mathbf{I} + \hat{\alpha}^{*2}\frac{\sigma_\delta^2}{1+\sigma_\delta^2}\mathbf{D}'^2_{\mathbf{x}}$, where $\mathbf{D}'_{\mathbf{x}}$ is the diagonal matrix with the elements of $\mathbf{x}_{\text{meas}}$ on the diagonal. Note that since $\boldsymbol{\Sigma}'_e$ has a diagonal covariance structure, calculation of the likelihood (11) is fast.

In order to ascertain that λ selection and the estimation of σ_δ^2 by the aforementioned functions worked properly and that both calculations are necessary elements of the P-spline model, two separate tests were performed during the validation study. First a P-spline model was fitted on the 500 simulated datasets

that assumed that there was no measurement error in PA (i.e. $\sigma_\delta^2 = 0$). When compared with the results of the P-spline model where σ_δ^2 had been estimated, the former model showed to have a larger error and bias in the estimation of average RMR (Fig. S1-1A,B). The fact that nevertheless a small bias remained using the second approach demonstrates that (11) is not an unbiased estimator of σ_δ^2. Second, an ordinary spline model was fitted on the 500 synthetic datasets that did not include a penalisation term ($\lambda = 0$). When compared with the performance of the penalised spline model, the model without penalisation had considerably larger estimation error in the time-dependent RMR (Fig. S1-1C). This shows that λ selection prevents overfitting and is therefore important for estimating the time variations in the RMR.

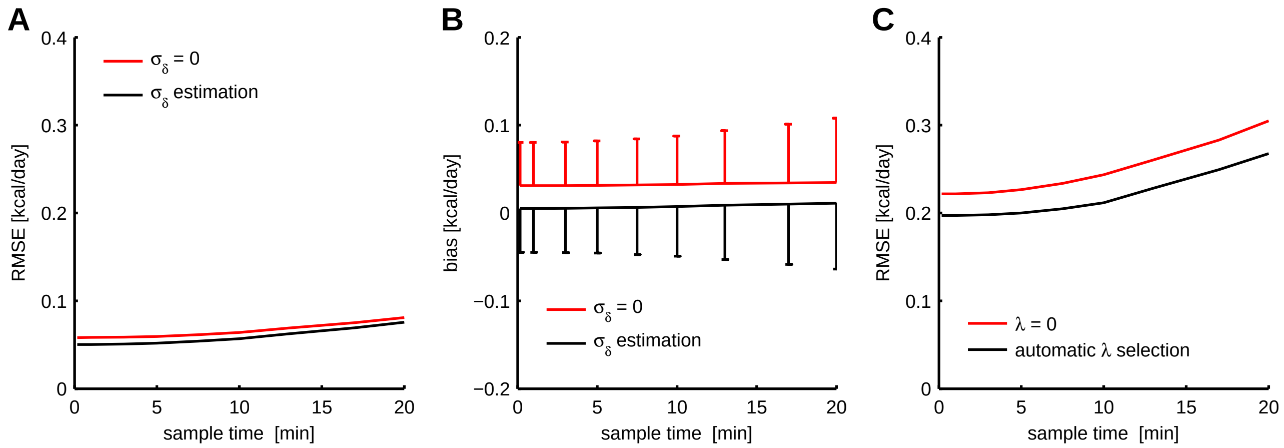

Figure S1-1. Design choices for the penalised spline regression model. Measurement error in physical activity, due to sensor noise and variability in the caloric cost of activity, caused the estimate of the average RMR to be biased because of regression dilution (**A** – **B**; red line). When the variance σ_δ^2 in the measurement error was estimated and taken into account by the corrected expression for estimation of the cost of activity, the bias was reduced from 0.032 kcal/day to 0.007 kcal/day (**B**). Estimating the time-dependent RMR by means of penalised splines (**C**; black line) as opposed to ordinary splines without penalisation (**C**; red line) was found necessary to prevent overfitting. The first order derivative of the splines function was penalised, and the smoothing parameter λ was selected by means of Generalised Cross Validation.

References

[1] Fuller WA (1987) Measurement Error Models. New York: Wiley.

[2] Nakamura T (1990) Corrected score function for errors-in-variables models: Methodology and application to generalized linear models. Biometrika 77: 127–137.

[3] Zhong XP, Fung WK, Wei BC (2002) Estimation in linear models with random effects and errors-in-variables. Ann Inst Statist Math 54: 595-606.

[4] Akaike H (1974) A new look at the statistical model identification. IEEE Trans Automat Control 19: 716–723.

[5] Eilers PHC, Marx BD (1996) Flexible smoothing with B-splines and penalties. Statist Sc 11: 89–121.

[6] Craven P, Wahba G (1979) Smoothing noisy data with spline functions: Estimating the correct degree of smoothing by the method of generalized cross-validation. Numer Math 31: 377-403.

[7] Ruppert D (2002) Selecting the number of knots for penalized splines. J Comp Graph Statist 11: 735–757.

[8] Ruppert D, Wand MP, Carroll RJ (2003) Semiparametric Regression. Cambridge: Cambridge University Press.
