# Supplementary material for: Estimation of Activity Related Energy Expenditure and Resting Metabolic Rate in Freely Moving Mice from Indirect Calorimetry Data
Source: PLoS One. 2012 May 4;7(5):e36162. doi: 10.1371/journal.pone.0036162 (PMC3344840; doi:10.1371/journal.pone.0036162)
Supplement: Text S2 — Characterisation of the relation between the measured physical activity and related energy expenditure. (PDF) [file pone.0036162.s002.pdf]

Supplemental Material Text S2

Estimation of Activity Related Energy Expenditure and Resting Metabolic Rate in Freely Moving Mice from Indirect Calorimetry Data

Jan Bert van Klinken[1], Sjoerd A.A. van den Berg, Louis M. Havekes, Ko Willems van Dijk

Characterisation of the PA–AEE relation

For the optimal estimation of the RMR and AEE, the relationship between the time-dependent activity measurements and the related energy expenditure must be linear. Therefore, before application of the P-spline regression model this relation needs to be characterised for the given activity sensor and indirect calorimetry system, such that a suitable preprocessing function can be constructed for the activity data.

As discussed in the main article, preprocessing of the activity data consists at least of the following two steps, which permit to align the energy expenditure and activity data: 1.) application of a low pass filter to mimic the gas diffusion effects, and 2.) downsampling of the convolved activity data to the sample time of the TEE. In addition, a mathematical function must be chosen that can rectify all existing nonlinearities between the measured activity and the related energy expenditure. In our experience, the choice of this function cannot be done automatically but must instead be based on careful inspection of the scatterplot of the TEE and the preprocessed activity data. In contrast, selection of the parameters of the preprocessing function can be performed automatically, namely by minimising the residual sum of squares of the P-spline model. In the case that indirect calorimetry and activity datasets of multiple subjects are analysed, then parameter selection must be based on minimisation of the total residual sum of squares of all subjects.

In the remainder of this supplementary text we discuss how we determined the preprocessing function for our data.

[1]E-mail: J.B.van_Klinken@lumc.nl

Estimation of the washout time of the metabolic chamber system The time variation in the respiratory exchange as it is measured at the level of the gas sensors does not reflect the instantaneous energy expenditure (i.e. at the level of the cell) but is distorted by the gas diffusion through the body and the chamber. We propose to model the gas diffusion effects by means of two linear compartments that have washout times τ_1 and τ_2, which represent the body and chamber respectively. In addition, we account for the delay τ_3 that is introduced by the tubing and gas dryers that are located between the chamber and gas sensors. See the Methods section in the main article for details.

We based the choice of τ_1 on the work of Even *et al.* [1], who proposed a value of $\tau_1 = 15$ s for rats, which was assumed to be independent of their body weight. Since mice are an order of a magnitude smaller than rats, we took a value of $\tau_1 = 1.5$ s for mice.

To assess the correctness of the linear diffusion model for our metabolic chamber system, we performed a separate experiment in which we saturated a single metabolic chamber with a gas mixture that had a CO_2 concentration of 0.5%. Subsequently the reference (ambient) air was switched back to the input airflow of the chamber and the CO_2 concentration in the outgoing air was measured for a period of 25 min with a sample time of 10 s. The result is shown in Fig. S2-1. Fitting a negative exponential function to the registered data yielded an estimate of $\tau_2 = 290$ s, which was close to the theoretical value of $\tau_2 = V/f = 293$ s that was based on the volume of the chamber ($V = 104\text{mm} \times 126\text{mm} \times 201\text{mm} = 2.63$ l) and the air flow ($f = 8.97$ ml/s). Hence, in the remainder of our study we calculated τ_2 as the volume of the cage divided by the air flow.

For the time delay we assumed a value of $\tau_3 = 5$ s, which was an estimate based on the flow rate and the length and diameter of the air tubes.

Linearisation of the PA-AEE relation In order to characterise the PA-AEE relation for our system we used the high time resolution metabolic chamber dataset (single mouse). From inspection of the scatterplot of the TEE versus the PA adjusted for gas diffusion effects, it followed that this relationship was strongly nonlinear for our metabolic chamber system (Fig. S2-2A,D). First, we investigated whether a power function x^p captured the nonlinearity in the PA-AEE relation well enough, where the value of p was selected by minimising the residual sum of squares of the P-spline model.

From the resulting scatterplot (Fig. S2-2B,E) it followed that the preprocessed PA indeed correlated better with the TEE ($r^2 = 0.82$) than did the

linear model ($r^2 = 0.58$). However, close inspection of the scatterplot showed that results were not optimal since there was still a nonlinear trend perceivable in the residuals. In addition it was observed that for low TEE the preprocessed PA was relatively high, which could be explained by the fact that the initial steepness of the power function had amplified the noise on the PA signal that occurred during periods of rest of the animal (i.e. when TEE is low). This type of noise can be attributed to unconscious movements such as those associated with respiration and was not counted as PA. Therefore, we extended the preprocessing function with a threshold to eliminate this noise from the activity data. Moreover, we applied a Gaussian kernel to smooth the raw activity data, which served to attenuate the noise that arises when infrared beam interruptions are collected in relatively short time bins (i.e. T_{PA} is low). The total preprocessing function that we used was

$$\mathrm{PA}(t) = R\,(\ \mathrm{PA}_{\mathrm{raw}}(t) * K_\sigma(t) - \theta\ )^p \tag{1}$$

with $\mathrm{PA}_{\mathrm{raw}}(t)$ the raw activity data expressed in counts/min, $\mathrm{PA}(t)$ the preprocessed activity data, $*$ the convolution operator, $K_\sigma(t)$ the Gaussian kernel function $K_\sigma(t) = \frac{1}{\sigma\sqrt{2\pi}}\exp\left(-\frac{t^2}{2\sigma^2}\right)$, p the power, and R the ramp function with threshold θ, defined as $R(x-\theta) = x-\theta$ if $x \geq \theta$ and $R(x-\theta) = 0$ otherwise. Subsequently, the preprocessed activity data was convolved with the impulse response $h_{\mathrm{delay}}(t)$ of the system/gas diffusion delay and was downsampled to the sample time of the TEE [2]

$$\mathrm{PA}'(\mathbf{t}_{\mathrm{TEE}}) = \mathrm{PA}(\mathbf{t}_{\mathrm{PA}}) * h_{\mathrm{delay}}(\mathbf{t}_{\mathrm{PA}})|_{\mathbf{t}_{\mathrm{PA}} \to \mathbf{t}_{\mathrm{TEE}}} \tag{2}$$

where $\mathrm{PA}'(\mathbf{t}_{\mathrm{TEE}})$ is a measure of the time-dependent AEE as it is measured at the sensor level $\mathrm{AEE}(\mathbf{t}_{\mathrm{TEE}}) = \alpha\ \mathrm{PA}'(\mathbf{t}_{\mathrm{TEE}})$, with α the caloric cost of activity (see also Eq. (4) of the main article).

We estimated the parameter set (σ, θ, p) of the preprocessing function (1) by minimising the residual sum of squares of the P-spline regression model. The optimal parameter values that we found were $\sigma = 26$ s, $\theta = 10.6$ counts/min and $p = 0.13$. Inspection of the resulting scatterplot shows that the preprocessing function had been successful in eliminating nonlinear trends and in adjusting the threshold (Fig. S2-2C,F). In addition, the strength of the correlation had improved ($r = 0.85$) with respect to using only the power function as preprocessing function.

[2]It is important to note that convolution with $h_{\mathrm{delay}}(t)$ must only be performed in the case that the measured TEE has not already been compensated for the gas diffusion effect. Such corrections are standardly performed for human indirect calorimetry data, where the filtering effect of gas diffusion is much larger because of the larger metabolic chambers.

A practical adjustment that was made to improve the time efficiency of the optimisation function was to set the penalisation coefficient and the PA error of the P-spline model that was used for optimisation to zero $\lambda = 0, \;\; \sigma_\delta = 0$. In order to prevent overfitting, the number of knots in the spline function that was used for estimating the preprocessing parameters was chosen slightly lower (10 knots/day). The preprocessing function and the optimisation routine are part of the TEE decomposition toolbox for MATLAB that can be obtained upon request.

Influence of the sample time of the preprocessing parameters We investigated whether the sample time T_{PA} was influencing the way in which PA should be preprocessed, by estimating the optimal value of p for different sample times. Lower resolution datasets of TEE were created by taking every N-th sample of the high resolution dataset, corresponding thus to a sample time T_{TEE} of $10N$ seconds. Lower resolution datasets for PA were created by adding the previous $N-1$ samples to each N-th sample, since beam breaks were reported in a cumulative fashion by our metabolic chamber system. It was found that the optimal order p_{opt} of the power function increased monotonically for longer T_{PA} (Fig. S2-3). An empirical function $p_{\mathrm{opt}}(T_{\mathrm{PA}}) = \frac{1.51}{2.0+\exp(T_{\mathrm{PA}}/6.1)} - 0.38$ of the optimal order was found to fit the data best, with T_{PA} the sample time in minutes.

Selection of the preprocessing parameters for the case study data For the low resolution dataset of the case study ($T_{\mathrm{PA}} = 60$ s, $T_{\mathrm{TEE}} = 7.5$ min) the optimal root order and threshold level were fitted by minimising the total residual sum of squares of all 15 mice. The optimal parameter values that were found were $p = 0.20$ and $\theta = 8.5$ counts/min; the kernel width σ was kept fixed at 26 s.

References

[1] Even PC, Perrier E, Aucouturier JL, Nicolaidis S (1991) Utilisation of the method of Kalman filtering for performing the on-line computation of background metabolism in the free-moving, free-feeding rat. Physiol Behav 49: 177–187.

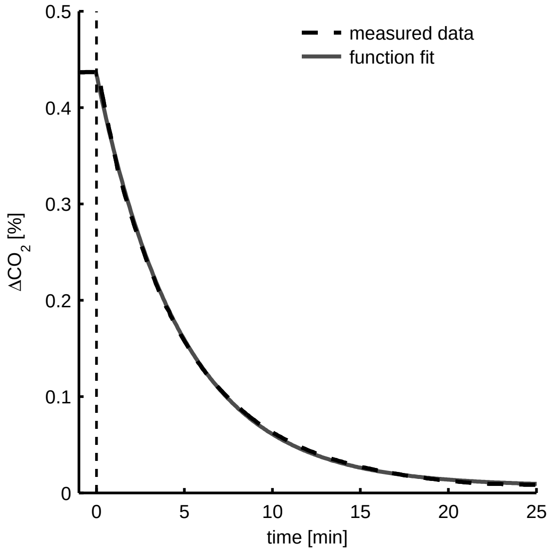

Figure S2-1. Determination of the chamber washout time τ_2. The effect of gas diffusion through the metabolic chamber on the measured respiratory exchange was evaluated by measuring the response in the CO_2 concentration on a step change in the composition of the inflowing air. A negative exponential function was fit to the step response and showed to describe the measured data very well. The washout time of the fitted function was $\tau_2 = 290$ s, which was close to the theoretical value of 293 s based on the volume of the chamber and the flow rate.

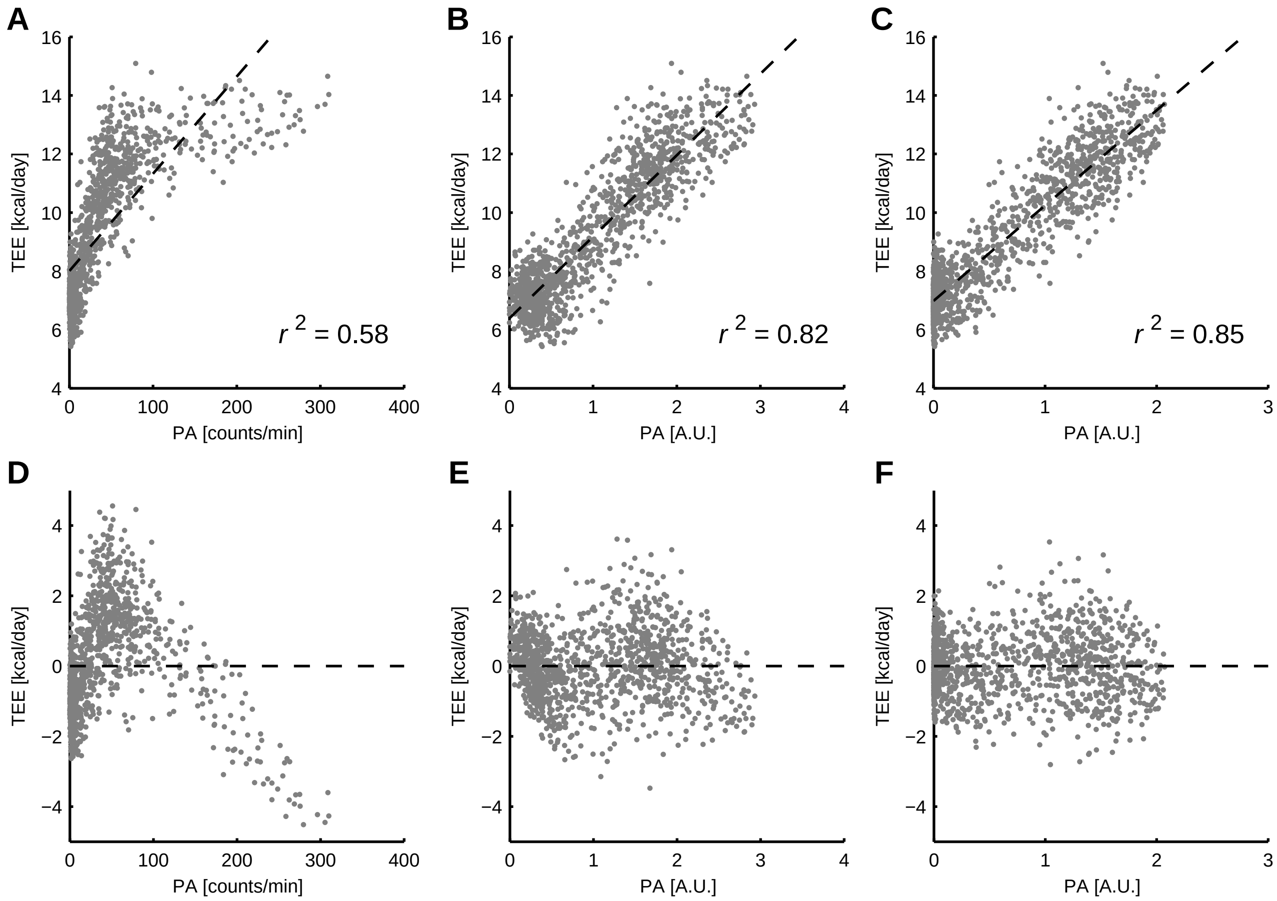

Figure S2-2. Nonlinearity in the PA–AEE relation. A scatterplot of the TEE versus the raw PA measurements shows that their relation is strongly nonlinear for the CLAMS metabolic chamber system (**A**). This also follows from the residuals of a linear regression model (**D**). Preprocessing PA with a power function notably improved the fit of the linear model (**B**), but still left a faint S-shape in the residuals (**E**). Additional smoothing and thresholding corrected for the amplification of noise during rest and further improved the fit of the linear model (**C**), also leaving no trends perceivable in the residuals (**F**).

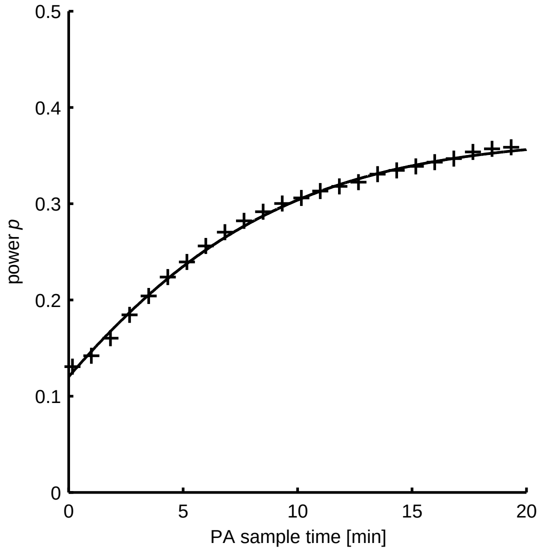

Figure S2-3. Dependency of the optimal power for preprocessing PA on T_{PA}. The optimal value for the power p for preprocessing the PA in the high resolution dataset increased monotonically for longer sample times T_{PA} (crosses). The black line gives the fitted empirical function that indicates the dependency of p_{opt} on the sample time: $p_{\mathrm{opt}}(T_{\mathrm{PA}}) = \frac{1.51}{2.0+\exp(T_{\mathrm{PA}}/6.1)} - 0.38$, with T_{PA} in minutes.
