# Supplementary material for: Estimation of Activity Related Energy Expenditure and Resting Metabolic Rate in Freely Moving Mice from Indirect Calorimetry Data
Source: PLoS One. 2012 May 4;7(5):e36162. doi: 10.1371/journal.pone.0036162 (PMC3344840; doi:10.1371/journal.pone.0036162)
Supplement: Text S3 — Detailed discussion of the conventional methods for estimating AEE and RMR: linear regression, averaging TEE for periods of zero activity and Kalman filtering. (PDF) [file pone.0036162.s003.pdf]

Supplemental Material Text S3

Estimation of Activity Related Energy Expenditure and Resting Metabolic Rate in Freely Moving Mice from Indirect Calorimetry Data

Jan Bert van Klinken[1], Sjoerd A.A. van den Berg, Louis M. Havekes, Ko Willems van Dijk

Conventional methods for estimating AEE and RMR

In this section we discuss the implementation of the TEE decomposition methods whose performance has been compared to that of the penalised spline regression model.

Linear regression. The traditional approach to determine the contribution of activity to TEE is to regress the sampled values of TEE against the measured intensity of PA [1–3]. The y-intercept of the regression line then corresponds to the average RMR and the slope corresponds to the average caloric cost of activity.

We implemented the linear regression model taking into account the delay induced by the gas diffusion in the metabolic chamber. That is, we correlated the measured TEE time sequence with the PA sequence convolved with h_{delay}

$$\text{TEE}(\mathbf{t}_{\text{TEE}}) = \mathbf{1}_n \mu_{\text{RMR}} + \alpha\, \mathbf{H}\, \text{PA}(\mathbf{t}_{\text{PA}}) + \boldsymbol{\varepsilon}$$

with $\mathbf{1}_n$ the $n \times 1$ vector with ones, μ_{RMR} the average RMR, and other variables as described in the Methods Section of the main article. Hence, the RMR is assumed to be constant in time: $\text{RMR}(\mathbf{t}_{\text{TEE}}) = \mathbf{1}_n \mu_{\text{RMR}}$. Assuming that the error vector $\boldsymbol{\varepsilon}$ is normal, zero mean and independent and identically distributed, the regression coefficients were estimated by ordinary least squares

$$\begin{bmatrix} \hat{\mu}_{\text{RMR}} \\ \hat{\alpha} \end{bmatrix} = (\mathbf{X}^{\text{T}}\mathbf{X})^{-1}\mathbf{X}^{\text{T}}\, \text{TEE}(\mathbf{t}_{\text{TEE}})$$

with the design matrix $\mathbf{X} = [\ \mathbf{1}_n\ ,\ \mathbf{H}\, \text{PA}(\mathbf{t}_{\text{PA}})\ ]$.

[1]E-mail: J.B.van_Klinken@lumc.nl

Averaging of TEE for zero activity. Another frequently used approach is to determine the RMR by averaging the TEE over periods in which physical activity is minimal [4, 5]. As for the previous method, this approach only estimates the average RMR:

$$\hat{\mu}_{\text{RMR}} = (\mathbf{z}^{\text{T}}\mathbf{z})^{-1}\mathbf{z}^{\text{T}}\ \text{TEE}(\mathbf{t}_{\text{TEE}})$$

with $\mathbf{z}$ the $n \times 1$ vector that contains ones for the time points for which the activity is sufficiently low and zeros otherwise. The criterion used in our study for zero activity was when $\mathbf{H}\ \text{PA}(\mathbf{t}_{\text{PA}})$ dropped below 5% of the average PA intensity. The cost of activity was subsequently calculated by dividing the difference of average TEE and $\hat{\mu}_{\text{RMR}}$ by the average amount of PA.

Kalman Filtering. In contrast to the former two methods, which do not take the time-dependency of the RMR into account, Even proposed to model the RMR as a random process that varies with time [6]. The relation between the RMR, CCA, AEE and TEE and the diffusion effects of the gas exchange are expressed as a state space model

$$\begin{aligned}
\text{RMR}_{i+1} &= \text{RMR}_i + \eta_{\text{RMR}} \\
\text{CCA}_{i+1} &= \text{CCA}_i + \eta_{\text{CCA}} \\
\text{AEE}_{i+1} &= \left(1 - \tfrac{T}{\tau_1}\right)\text{AEE}_i + \tfrac{T}{\tau_1}\text{PA}_i \cdot \text{CCA}_i + \eta_{\text{AEE}} \\
\text{TEE}'_{i+1} &= \left(1 - \tfrac{T}{\tau_2}\right)\text{TEE}'_i + \tfrac{T}{\tau_2}\text{RMR}_i + \tfrac{T}{\tau_2}\text{AEE}_i + \eta_{\text{TEE}} \\
\text{TEE}_i &= \text{TEE}'_i + \varepsilon
\end{aligned}$$

That is, RMR and CCA are modelled as autoregressive random processes of order one, with η_{RMR} and η_{CCA} the process noises. The subscript i is shorthand notation for the time instance at which a measurement was taken. The diffusion effects of the gas exchange are approximated by first order discrete-time filters, with T the sample time and τ_1 and τ_2 the washout times of the subject and chamber volume as defined in the Methods Section of the main article. The term TEE' corresponds to the total energy expenditure at the outlet of the chamber without the sensor noise ε. The error terms η_{AEE} and η_{TEE} permit the AEE and TEE' to diverge from the prediction as based on the CCA, PA and RMR alone. Note that in this notation it is implicit that TEE and PA are sampled with the same sample time T and at the same time instances.

Estimates of the state vector, and hence of the RMR and AEE, can be obtained by means of Kalman filtering. Kalman filtering is a method of numerical filtering that works by recursively predicting the future state of the system and subsequently correcting it with the measured output of the system; for a

comprehensive discussion on the Kalman filter equations, please see [6, 7]. The performance of the Kalman filter depends on the choice of the initial state vector and on the (co)variances of the process noises η_{RMR}, η_{CCA}, η_{AEE}, η_{TEE} and the measurement noise ε. In general the process noise variances are unknown, and appropriate values must be found by manually tuning the Kalman filter [6].

Here we selected values for the process noises as to optimise the estimation accuracy of the Kalman filter of the average and time-dependent RMR on the simulated datasets. The initial state vector was determined by linear regression. Since the model equations describe the RMR at the level of the subject, in the validation study the time series were filtered with

$$h_{\mathrm{chamber}}(t) = \begin{cases} \frac{1}{\tau_2} \mathrm{e}^{-\frac{t}{\tau_2}} & t \geq 0 \\ 0 & t < 0 \end{cases}$$

In this way the RMR was obtained as it would be measured at the level of the outlet of the metabolic chamber, such that comparison with the results of the P-spline model was possible. An adjustment was made to the Kalman filter model equations by replacing the ratios $\frac{T}{\tau_1}$ and $\frac{T}{\tau_2}$ by the terms $1 - \exp\left(-\frac{T}{\tau_1}\right)$ and $1 - \exp\left(-\frac{T}{\tau_2}\right)$ respectively. The corrected terms provide more accurate time discretisations that guarantee impulse invariance of the filter [8], whereas the former are only accurate for low sample times (i.e. $\tau_1 \gg T$ and $\tau_2 \gg T$) and cause the filter to become unstable if these conditions are not being met.

References

[1] Ravussin E, Lillioja S, Anderson TE, Christin L, Bogardus C (1986) Determinants of 24-hour energy expenditure in man – methods and results using a respiratory chamber. J Clin Invest 78: 1568–1578.

[2] Kumahara H, Schutz Y, Ayabe M, Yoshioka M, Yoshitake Y, et al. (2004) The use of uniaxial accelerometry for the assessment of physical-activity-related energy expenditure: a validation study against whole-body indirect calorimetry. Br J Nutr 91: 235–243.

[3] Bjursell M, Gerdin AK, Lelliott CJ, Egecioglu E, Elmgren A, et al. (2008) Acutely reduced locomotor activity is a major contributor to Western diet-induced obesity in mice. Am J Physiol Endocrinol Metab 294: 251–260.

[4] Nonogaki K, Abdallah L, Goulding EH, Bonasera SJ, Tecott LH (2003) Hyperactivity and reduced energy cost of physical activity in serotonin 5-HT(2C) receptor mutant mice. Diabetes 52: 315-320.

[5] Novak CM, Kotz CM, Levine JA (2006) Central orexin sensitivity, physical activity, and obesity in diet-induced obese and diet-resistant rats. Am J Physiol Endocrinol Metab 290: E396–403.

[6] Even PC, Perrier E, Aucouturier JL, Nicolaidis S (1991) Utilisation of the method of Kalman filtering for performing the on-line computation of background metabolism in the free-moving, free-feeding rat. Physiol Behav 49: 177–187.

[7] Haykin S (2002) Adaptive Filter Theory. Upper Saddle River: Prentice Hall, 4th edition.

[8] Oppenheim AV, Schafer RW, Buck JR (1999) Discrete-time signal processing. Upper Saddle River: Prentice Hall.
