# Supplementary material for: Estimation of Activity Related Energy Expenditure and Resting Metabolic Rate in Freely Moving Mice from Indirect Calorimetry Data
Source: PLoS One. 2012 May 4;7(5):e36162. doi: 10.1371/journal.pone.0036162 (PMC3344840; doi:10.1371/journal.pone.0036162)
Supplement: Text S4 — Simulation protocol of indirect calorimetry data. (PDF) [file pone.0036162.s004.pdf]

Supplemental Material Text S4

Estimation of Activity Related Energy Expenditure and Resting Metabolic Rate in Freely Moving Mice from Indirect Calorimetry Data

Jan Bert van Klinken[1], Sjoerd A.A. van den Berg, Louis M. Havekes, Ko Willems van Dijk

Simulation of indirect calorimetry data

We here give a detailed account of how the simulated metabolic chamber data was generated. Spontaneous PA was modelled as a sequence of periods of uninterrupted activity (activity bouts) of varying duration and intensity, depending on the hour of the day. To ensure realistic results, simulation parameters were based on the metabolic chamber dataset from the case study. Visual inspection of the experimental data suggested that activity patterns were different between both groups of mice: the obese male mice were active for short periods of time and with short pauses, while the lean female mice had longer activity bouts that occurred with a lower frequency (Fig. S4-1). Quantitative analysis showed that there was a difference between groups in the amount of time mice were active, the PA intensity, and the bout frequency (Fig. S4-2). Therefore, we modelled activity patterns for both groups of mice separately, using the analysis results shown in Fig. S4-2 as benchmark.

Activity bouts were simulated to occur every 47 min for male mice and every 65 min for female mice; random variation in activity bout occurrence was introduced by multiplying the time a bout occurred with a normally distributed random variable $\varepsilon_{\mathrm{T\ bouts}} \sim \mathcal{N}(1, 0.09)$ (normal distributions are denoted as $\mathcal{N}(\mu, \sigma^2)$, with μ the mean and σ^2 the variance). The length of an activity bout was modelled to vary between day and night according to a cosine function with a maximum of 27 min at 00:00h and a minimum of 10 min at 12:00h for male mice, and a maximum of 45 min at 00:00h and a minimum of 16 min at 12:00h for female mice. Random variation was simulated by multiplying the bout length with a normally distributed random variable $\varepsilon_{\mathrm{bout\ length}} \sim \mathcal{N}(1, 0.09)$. Relative intensity of PA was simulated to vary between night and day with a

[1]E-mail: J.B.van_Klinken@lumc.nl

cosine function with an amplitude of 0.09 and an offset of 1; random variation was introduced by multiplying the amplitude of each bout with a normally distributed random variable $\varepsilon_{\mathrm{PA\ intensity}} \sim \mathcal{N}(1, 0.005)$. Instantaneous AEE was derived from the PA signal by scaling it such that the average AEE amounted to 2.0 kcal/day for both groups (Tab. 1); consequently, the relation between AEE and PA was linear for the simulated data. The intensity of activity as it was measured by the infrared beam monitors was simulated by scaling the activity function to a time-dependent probability density function that was used to sample the beam break occurrences. It was assumed that 39000 beam breaks occurred per day for male mice and 50000 for female mice, which were binned into 10 second intervals, corresponding thus to a sample time of PA of 10 seconds. Additional PA measurement error due to variations in the caloric cost of activity was simulated by first multiplying the amplitude of each activity bout with a normally distributed random variable $\varepsilon_{\mathrm{CCA}} \sim \mathcal{N}(1, 0.01)$.

To ascertain that the simulated activity patterns resembled experimental data, 500 datasets with activity patterns of male and female mice were generated separately from the main validation study, and the amount of time that mice were active, the PA intensity, and the bout frequency were calculated in the same way as was done for the data from the case study (Fig. S4-2). Comparison of the results showed that these parameters were equal for experimental and simulated data. Moreover, visual comparison of the activity patterns confirmed that the simulated data were reasonably similar to the experimental data (Fig. S4-1). In the main validation study, which served to evaluate the accuracy of TEE decomposition, the parameters concerning activity patterns were varied linearly between those from both groups of mice such that the simulated data reflected a mixture of male and female mice. The simulated random variation in caloric cost of activity $\varepsilon_{\mathrm{CCA}}$ had been chosen such that the estimated standard deviation of PA measurement error $\hat{\sigma}_\delta$ on experimental data (0.061 ± 0.054) was close to that of the simulated data (0.065 ± 0.032).

Simulation parameters of the RMR time series and the TEE measurement noise were based on the high resolution experimental dataset. In order to simulate realistic time-dependent fluctuations in the RMR, we investigated the time sequence consisting of the estimated RMR and the residuals resulting from the P-spline regression model. Inspection of this signal showed that there was considerable ultradian variation present that did not correlate with PA. Part of these fluctuations reflected the fast changes that occur in RMR due to endogenous (nervous or hormonal) changes [1], while the remainder was due to measurement noise; i.e. noise in the gas sensors and fluctuations associated with

non-homogeneous mixing of expired air in the metabolic chamber. Since most of the high frequency fluctuations observed in the residuals could not have been caused by fluctuations in the RMR as these would have been filtered out by the gas diffusion effect, we assumed that these were due to measurement noise. The instantaneous RMR and the measurement noise signal were modelled as white Gaussian processes filtered respectively by the linear filters

$$H_{\text{RMR inst.}}(s) = 40\tfrac{1+135s}{(1+800s)(1+4s)(1+3s)}$$
$$H_{\text{noise}}(s) = 4\tfrac{1}{(1+4s)(1+0.3s)}$$

with H the transfer function in the frequency domain and s the complex angular frequency expressed in rad min^{-1}. The zeros and poles of both transfer functions were chosen such that the modelled power spectral density (PSD) of the RMR plus the measurement noise

$$\text{PSD}_{\text{RMR+noise}} = H^2_{\text{delay}}(s) \cdot H^2_{\text{RMR inst.}}(s) + H^2_{\text{noise}}(s)$$

with

$$H_{\text{delay}}(s) = \frac{1}{(1+\tau_1 s)(1+\tau_2 s)}$$

corresponded to the PSD of the time sequence of the estimated RMR plus the residuals (Fig. S4-3).

Given the sample time of $T = 10$ s, the continuous time filters were transformed into discrete time filters by means of the impulse invariance approach [2], which permitted to generate synthetic time sequences of the measurement noise and instantaneous RMR. In addition, a constant value of 10.0 kcal/day was added to the RMR time sequence plus a cosine function of a 24 hour period and 1.0 kcal/day peak-to-peak amplitude to mimic the day-night rhythm (Tab. 1). The measured TEE time sequence was calculated by adding the instantaneous RMR and AEE time sequence and convolving it with h_{delay} (the washout and delay times were set to $\tau_1 = 1.5$ s, $\tau_2 = 300$ s and $\tau_3 = 0$ s) and by subsequently adding the time sequence representing the TEE measurement noise.

References

[1] Even PC, Perrier E, Aucouturier JL, Nicolaidis S (1991) Utilisation of the method of Kalman filtering for performing the on-line computation of background metabolism in the free-moving, free-feeding rat. Physiol Behav 49: 177–187.

[2] Oppenheim AV, Schafer RW, Buck JR (1999) Discrete-time signal processing. Upper Saddle River: Prentice Hall.

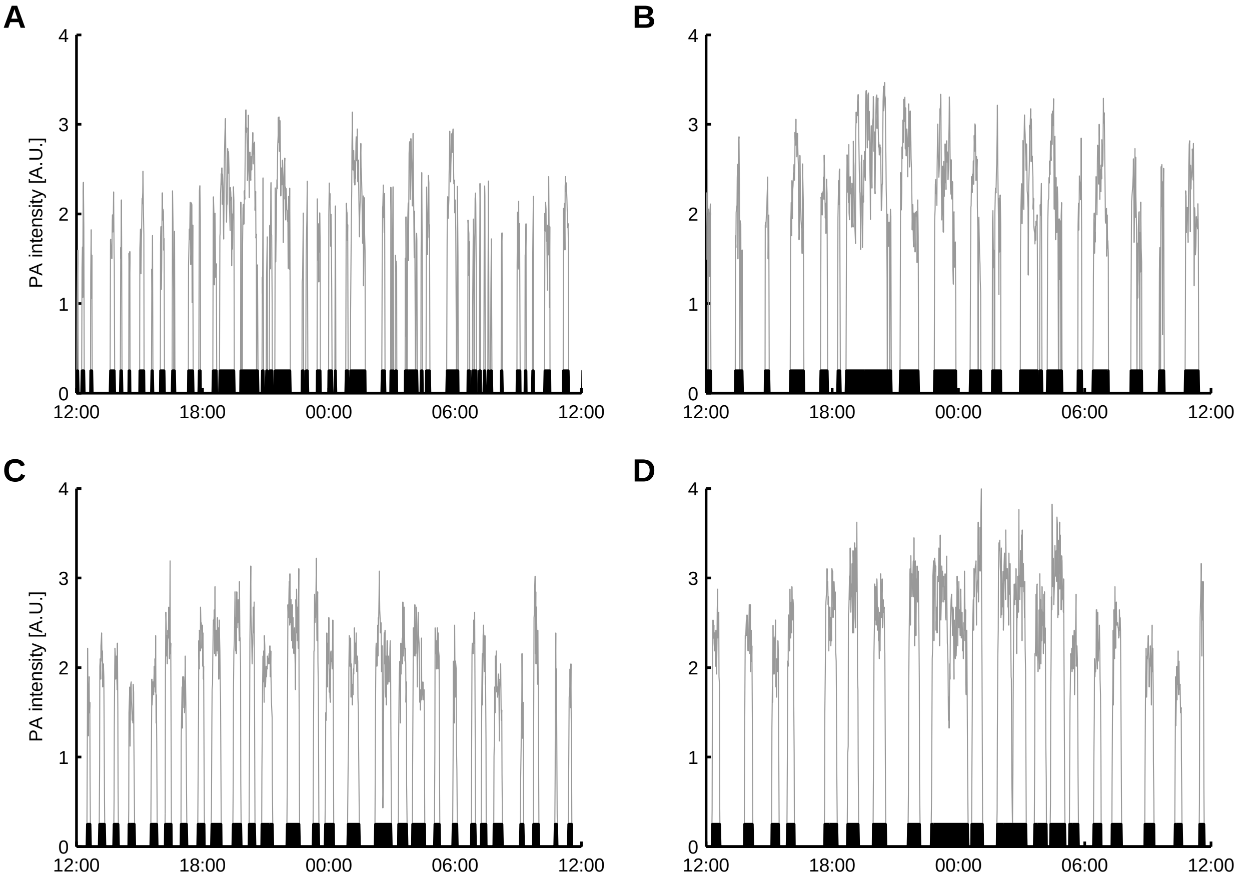

Figure S4-1. Simulation of activity patterns. A difference was found in activity patterns between the obese male (**A**) and lean female mice (**B**) in the case study: male mice were active for short periods of time and had short pauses, while female mice had longer periods of activity and rest. Gray lines indicate the preprocessed physical activity, black lines indicate the activity bouts. Activity bouts were detected by requiring a minimal duration of activity of 2 minutes and a minimal rest period of 3 minutes; moreover, the 5% of smallest activity bouts were not considered. Simulated activity patterns were based on the case study data, and showed to realistically mimic the behaviour of the male (**C**) and female mice (**D**).

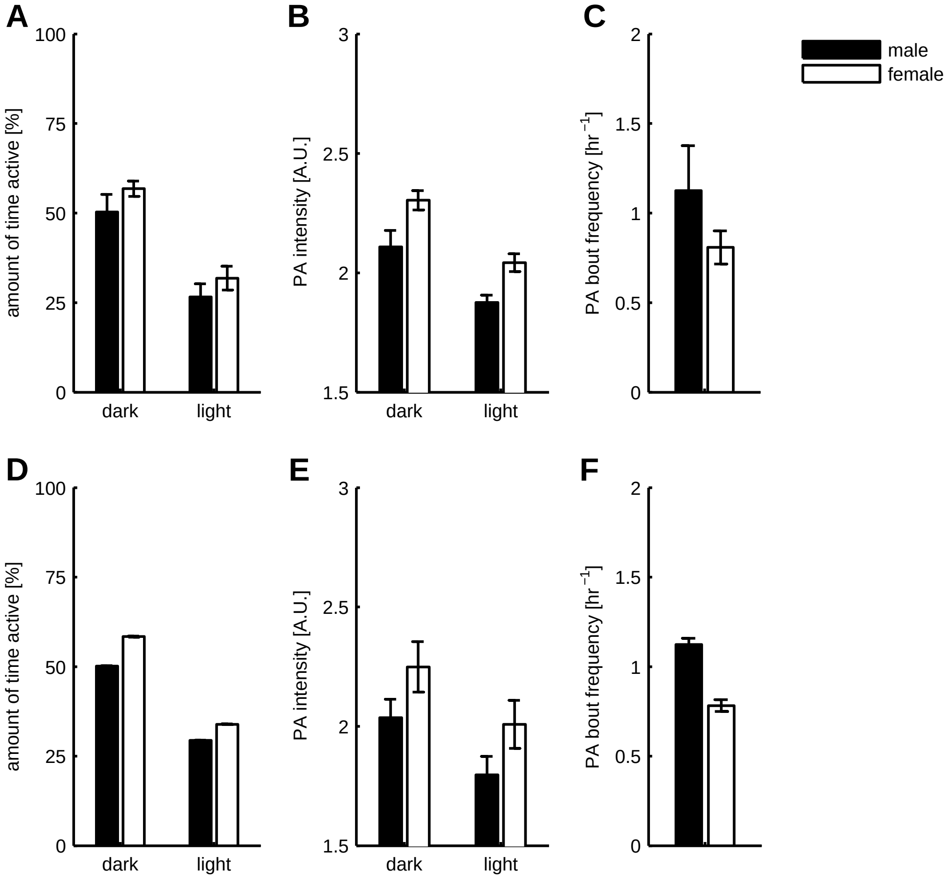

Figure S4-2. Characterisation of activity patterns. Activity patterns of both groups of mice from the case study were analysed quantitatively to provide a benchmark for fitting the parameters of the simulation data. Female mice were active for more time than male mice during both night and day (**A**), and were also more intensely active (**B**); error bars indicate standard deviation. In addition, activity bouts in female mice occurred with a lower frequency than in male mice (**C**). Simulation parameters were fit as to resemble the experimental data (**D** – **F**).

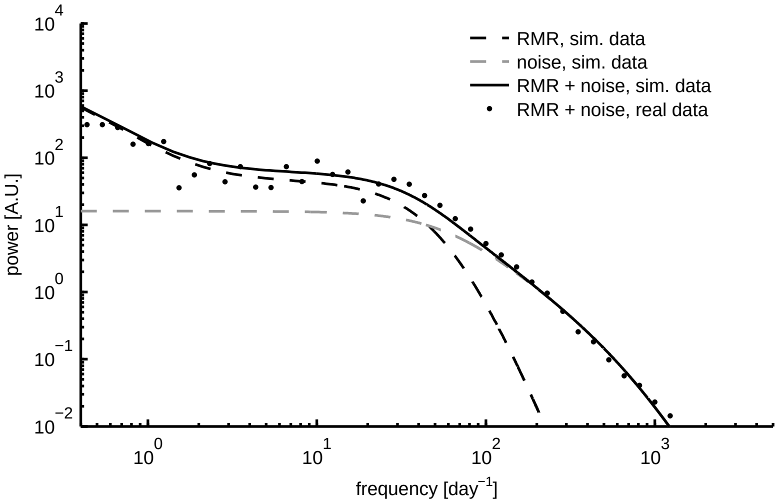

Figure S4-3. Power spectral density of RMR and TEE measurement noise. The time sequence of the estimated RMR plus the residuals, as calculated from the high resolution metabolic chamber dataset with the P-spline regression model, was used to determine the power spectral density of the RMR signal and the noise in the TEE measurements. This spectrum served to fit the parameters of the transfer functions that were used to generate synthetic time sequences of the RMR and the measurement noise in the validation study.
