# Supplementary material for: Estimation of Activity Related Energy Expenditure and Resting Metabolic Rate in Freely Moving Mice from Indirect Calorimetry Data
Source: PLoS One. 2012 May 4;7(5):e36162. doi: 10.1371/journal.pone.0036162 (PMC3344840; doi:10.1371/journal.pone.0036162)
Supplement: Text S5 — Calculation of the downsampling induced variability. (PDF) [file pone.0036162.s005.pdf]

Supplemental Material Text S5

Estimation of Activity Related Energy Expenditure and Resting Metabolic Rate in Freely Moving Mice from Indirect Calorimetry Data

Jan Bert van Klinken[1], Sjoerd A.A. van den Berg, Louis M. Havekes, Ko Willems van Dijk

Downsampling induced variability

We here propose downsampling induced variability (DIV) as a measure of the robustness of a TEE decomposition method to low sample rates. We define DIV as the variability between RMR estimates from a range of low time resolution datasets that have been extracted from a single high resolution dataset by means of downsampling. In the following paragraphs we describe how the DIV is calculated for the average and time-dependent RMR and demonstrate that it is always smaller than the total root mean square estimation error, thus providing a bias-variance decomposition of the estimation error.

We first show the bias-variance decomposition for the estimation error in the average RMR. Let a TEE time series of n datapoints be downsampled by a factor N into N separate datasets in the following way. The j-th downsampled dataset TEE_j^N contains the samples $\mathrm{TEE}_j^N[i] = \mathrm{TEE}[Ni + j]$, where the index i ranges from 0 to $\lfloor \frac{n-j}{N} \rfloor$ and the dataset number j ranges from 1 to N, with $\lfloor \cdot \rfloor$ the floor function and the square brackets [ ] indicating the position within a vector. As a result, from a single TEE dataset sampled with a sample time T_{TEE}, N separate datasets are created with an effective sample time of N times T_{TEE}. Now let $\hat{\mu}_j$ be the estimate of the average RMR as based on the j-th downsampled dataset TEE_j^N, let $\bar{\mu}$ be the mean of all N average RMR estimates $\bar{\mu} = \frac{1}{N} \sum_{j=1}^{N} \hat{\mu}_j$, and let μ be the true average RMR which is unknown for experimental data. Then the total mean square error (MSE) of the average RMR estimate is decomposed as follows

[1]E-mail: J.B.van_Klinken@lumc.nl

$$
\begin{aligned}
\mathrm{MSE}_{\mathrm{av.}} &= \frac{1}{N}\sum_{j=1}^{N}(\hat{\mu}_j-\mu)^2 \\
&= \frac{1}{N}\sum_{j=1}^{N}((\hat{\mu}_j-\bar{\mu})-(\mu-\bar{\mu}))^2 \\
&= \frac{1}{N}\sum_{j=1}^{N}(\hat{\mu}_j-\bar{\mu})^2 \;+\; (\mu-\bar{\mu})^2 \;-\; \frac{2}{N}\sum_{j=1}^{N}(\hat{\mu}_j-\bar{\mu})\,(\mu-\bar{\mu}) \qquad (1)\\
&\qquad\text{variance} \quad + \quad \text{bias} \quad + \qquad\qquad 0
\end{aligned}
$$

Expression (1) corresponds to the classical decomposition of the total mean square error into a bias and variance term. More specifically regarding to TEE decomposition, the variance component is a measure of the error that is introduced into average RMR estimation due to downsampling with a factor N, which can be interpreted as the error term due to sampling with sample time $N \cdot T_{\mathrm{TEE}}$ instead of T_{TEE}. Hence, we define the DIV for the average RMR at a given downsampling factor N as

$$
\mathrm{DIV}_{\mathrm{av.}} = \sqrt{\frac{1}{N}\sum_{j=1}^{N}(\hat{\mu}_j-\bar{\mu})^2} \qquad (2)
$$

Importantly, since the variance term is only based on the set of estimates of the average RMR, but not on the actual average RMR, it can be determined from experimental data.

The same bias-variance decomposition generally does not exist for vectors. However, since the accuracy of the time-dependent RMR estimate is determined only for frequency components under 6 day^{-1}, a bias-variance decomposition can be defined that is based on the fact that RMR estimates from different datasets TEE_j^N should not change much between two succeeding time points. Formally, let RMR_j^N be the j-th estimated time sequence of RMR based on the downsampled dataset TEE_j^N, with j ranging from 1 to N. Since we are only interested in estimating the low frequency components in RMR, all frequencies above 6 day^{-1} are filtered out separately for each estimated time series RMR_j^N. The time instants for which the RMR is estimated are disparate between datasets RMR_j^N, so we can define a single composite RMR time series of size n that includes all datasets RMR_j^N in the same way as has been done for TEE. That is, let the composite time series $\mathrm{RMR}_{\mathrm{tot}}^N$ contain the samples $\mathrm{RMR}_{\mathrm{tot}}^N[Ni+j] = \mathrm{RMR}_j^N[i]$, where the index i ranges from 0 to $\lfloor \frac{n-j}{N} \rfloor$ and the dataset number j ranges from 1 to N.

We will now propose the bias-variance decomposition of the total mean square estimation error of the time-dependent RMR. To simplify notation, let $\mathbf{u}$ be the $n \times 1$ vector that contains the composite RMR time series $\mathrm{RMR}_{\mathrm{tot}}^{N}$ for downsampling factor N, and let $\mathbf{v}$ be the $n \times 1$ vector representing the true RMR time series for frequency components below 6 day^{-1}. Now let $\mathbf{F}$ be the $n \times n$ matrix that filters out all frequency components above 6 day^{-1} upon multiplication. That is, $\mathbf{F} = \mathbf{W}^{\mathrm{T}}\mathbf{S}\mathbf{W}$ with $\mathbf{W}$ the Discrete Fourier Transform matrix with elements $w_{ij} = \frac{1}{\sqrt{n}}\exp\left(-2\pi\sqrt{-1}\frac{(i-1)(j-1)}{n}\right)$ [1], and $\mathbf{S}$ the diagonal matrix with elements $s_{ii} = 1$ when $\cos(2\pi\frac{i-1}{n}) \geq \cos(2\pi T_{\mathrm{TEE}}\frac{6}{24\cdot 60})$, with T_{TEE} expressed in minutes, and $s_{ii} = 0$ otherwise. Note that $\mathbf{F}$ is symmetric ($\mathbf{F}^{\mathrm{T}} = \mathbf{F}$) and idempotent ($\mathbf{F}^2 = \mathbf{F}$). Since the true RMR time series are filtered, we have $\mathbf{v} = \mathbf{F}\mathbf{v}$. Now let $\mathbf{w} = \mathbf{F}\mathbf{u}$ be the filtered composite RMR time series; we then define the decomposition of the time-dependent RMR estimation error as

$$
\begin{aligned}
\mathrm{MSE}_{\mathrm{time-dep.}} &= \frac{1}{n}(\mathbf{u}-\mathbf{v})^{\mathrm{T}}(\mathbf{u}-\mathbf{v}) \\
&= \frac{1}{n}((\mathbf{u}-\mathbf{w})-(\mathbf{v}-\mathbf{w}))^{\mathrm{T}}((\mathbf{u}-\mathbf{w})-(\mathbf{v}-\mathbf{w})) \\
&= \frac{1}{n}(\mathbf{u}-\mathbf{w})^{\mathrm{T}}(\mathbf{u}-\mathbf{w}) + \frac{1}{n}(\mathbf{v}-\mathbf{w})^{\mathrm{T}}(\mathbf{v}-\mathbf{w}) - \frac{2}{n}(\mathbf{u}-\mathbf{w})^{\mathrm{T}}(\mathbf{v}-\mathbf{w}) \\
&\qquad\text{variance} \qquad + \qquad \text{bias} \qquad + \qquad 0
\end{aligned} \tag{3}
$$

Expression (3) shows that the total mean square estimation error of the time-dependent RMR can be decomposed into a variance term and a bias term. The fact that the third term in (3) is equal to zero follows directly from the properties of $\mathbf{F}$ and $\mathbf{v}$, namely $(\mathbf{u}-\mathbf{w})^{\mathrm{T}}(\mathbf{v}-\mathbf{w}) = \mathbf{u}^{\mathrm{T}}\left(\mathbf{F}-\mathbf{F}^{\mathrm{T}}\mathbf{F}\right)(\mathbf{v}-\mathbf{u}) = 0$.

The variance part in (3) is defined as the MSE of the difference between the composite RMR time series $\mathbf{u}$ and the filtered time series $\mathbf{w}$, and quantifies the variation in RMR between succeeding time points (i.e. from succeeding downsampled datasets). It equals the power in the frequency components in $\mathbf{u}$ above 6 day^{-1}. We define the DIV for the time-dependent RMR as

$$
\mathrm{DIV}_{\mathrm{time-dep.}} = \sqrt{\frac{1}{n}(\mathbf{u}-\mathbf{w})^{\mathrm{T}}(\mathbf{u}-\mathbf{w})} \tag{4}
$$

Importantly, since $\mathbf{u}$ and $\mathbf{w}$ depend only on the time-dependent RMR estimates, the variance term can be calculated from experimental data, i.e. in the case when the actual time-dependent RMR $\mathbf{v}$ is unknown. Note that $\mathrm{DIV}_{\mathrm{av.}}$ is a specific case of $\mathrm{DIV}_{\mathrm{time-dep.}}$; namely, $\mathrm{DIV}_{\mathrm{av.}}$ is equal to $\mathrm{DIV}_{\mathrm{time-dep.}}$ with $\mathbf{F}$ filtering out all frequencies above zero.

References

[1] Higham NJ (2008) Functions of Matrices: Theory and Computation. Philadelphia: SIAM.
